# The Roles of Discrete Populations of Neurons Expressing Short Neuropeptide F in Sleep Induction in *Drosophila melanogaster*

**DOI:** 10.1111/gbb.70010

**Published:** 2025-02-07

**Authors:** Jamie M. Stonemetz, Nikoleta Chantzi, Emily L. Perkins, Aaliyah J. Peralta, Debra R. Possidente, John P. Tagariello, Marryn M. Bennett, Hooralain Alnassar, Andrew M. Dacks, Christopher G. Vecsey

**Affiliations:** ^1^ Neuroscience Program Skidmore College Saratoga Springs New York USA; ^2^ Neuroscience Program Brandeis University Waltham Massachusetts USA; ^3^ Department of Computer Science Skidmore College Saratoga Springs New York USA; ^4^ Department of Biology West Virginia University Morgantown West Virginia USA; ^5^ Department of Neuroscience West Virginia University Morgantown West Virginia USA; ^6^ Department of Biology Case Western Reserve University Cleveland Ohio USA

**Keywords:** circadian clock, Cryptochrome, *Drosophila melanogaster*, optogenetic, short neuropeptide F (sNPF), sleep

## Abstract

Sleep is of vital importance in our lives, yet we are far from understanding the neuronal networks that control the amount and timing of sleep. There is substantial conservation of known sleep‐regulating transmitters, allowing for studies in simpler organisms to lead the way in gaining insight into the organization of sleep control circuits. In *Drosophila melanogaster* , we recently showed that optogenetic activation of neurons that produce the neuropeptide Y (NPY)‐related transmitter short neuropeptide F (sNPF) increases time spent asleep. However, sNPF is expressed in several neuronal populations, and thus it is unknown which of those populations play roles in the sleep‐promoting effect. In this study, we addressed this issue using a genetic approach to limit optogenetic activation to subsets of sNPF‐expressing neurons. We found that sleep promotion was shorter‐lived when cryptochrome (CRY)‐positive neurons were excluded from being activated. Pigment‐dispersing factor (PDF) neurons were not required for sleep promotion, nor were mushroom body (MB) neurons. Acute reactions to a short, 10‐s period of optogenetic activation were largely unchanged by excluding activation of the three neuronal populations mentioned above. Together, these results suggest that clock neurons that are CRY‐positive and PDF‐negative are important contributors to the long‐lasting sleep promotion produced by sNPF neuron activation. However, other neurons targeted by the sNPF‐GAL4 driver appear to mediate the more rapid behavioral responses. Future studies will seek to identify these additional sNPF neuron populations and to determine how sNPF‐expressing clock neurons act in concert with other neuronal circuits to promote sleep.

## Graphical Abstract

It is critical to understand how sleep is regulated. Short neuropeptide F (sNPF) neurons have been shown to regulate sleep in the fruit fly, *Drosophila melanogaster*. In this study, we found that a subset of sNPF neurons that express cryptochrome but not pigment dispersing factor play an important role in driving long‐lasting increases in sleep induced when sNPF neurons are activated.

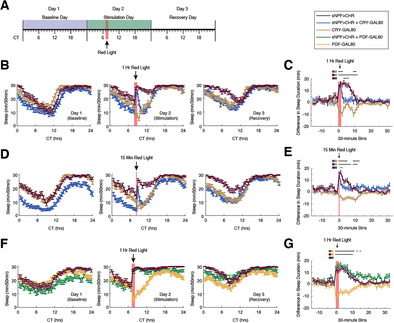

## 1 Introduction

Sleep is a widely conserved biological function, behaviorally defined as a period of quiescence with reduced sensory responsiveness that is regulated by a circadian clock as well as a homeostatic system [1, 2]. However, our understanding of how these brain networks interact to govern behavioral outputs is far from complete. Clinically, discovering how the timing and amount of sleep are controlled could allow for more effective treatments for sleep and circadian ailments, such as insomnia, hypersomnolence, narcolepsy, non‐24, delayed and advanced sleep phases, rapid eye movement (REM) behavior disorder, and others [3]. And at a more basic science level, gaining knowledge about sleep regulatory systems in diverse species can inform us about the evolution and functions of sleep [4].

Complex behaviors such as sleep involve the integration of a variety of internal and external cues in order to be properly regulated and benefit the survival of the organism. Sleep is not always a beneficial behavior, such as when predation risk is high or when it would be more advantageous to be searching for food or a mate. However, excessive sleep deprivation is associated with reduced health and even mortality in a variety of species [5, 6, 7]. Therefore, the sleep regulating centers of the brain must perform careful calculations to time sleep appropriately. Given the complexity of these neural calculations, it is beneficial to study organisms with simpler nervous systems to examine the principles underlying sleep regulation.

The fruit fly *Drosophila melanogaster* is one such model. *Drosophila* shows a resting behavior with the core characteristics of sleep, including reduced sensory responsiveness, a preferred location and body posture during sleep, altered brain activity, and regulation by both circadian and homeostatic influences [8, 9, 10, 11, 12, 13]. Several components of the neural circuitry underlying sleep regulation in *Drosophila* have been identified [2, 14] and molecular markers of sleep and wake have been characterized [15, 16, 17]. Additionally, a wide array of genetic tools can be readily used to monitor and manipulate the fruit fly nervous system, such as the GAL4/UAS system for controlled expression of transgenes within cells of interest [18, 19]. Together, these factors make *Drosophila melanogaster* an excellent organism in which to examine the cellular and molecular basis of sleep and circadian rhythms.

Circadian time‐keeping relies on molecular feedback loops to generate circadian oscillations within the cell by generating rhythmic gene expression and protein accumulation in the circadian clock cells of the brain's master biological clock. This mechanism is well conserved—all animals possess circadian clocks, which allow them to anticipate and prepare for the environmental changes that occur over the course of the day [20]. The circadian clock network in the central brain of *Drosophila* is composed of about 150 neurons, organized into clusters that include the small ventrolateral neurons (s‐LNvs), the large ventrolateral neurons (l‐LNvs), three classes of dorsal neurons (DN1, DN2, and DN3), and the dorsolateral neurons (LNds) [21, 22]. Morning and evening activity in *Drosophila* are governed by the LNvs and LNds, respectively [23, 24, 25], with the l‐LNvs being wake‐promoting while the exact role of the s‐LNvs are critical for maintaining rhythmicity in total darkness but whose role in sleep regulation is less well‐defined [21, 24]. Beyond the circadian clock neurons, the mushroom bodies, the fan‐shaped body, the pars intercerebralis, and the ellipsoid body all play roles in sleep regulation as well [21]. Communication among all of these brain regions is likely to allow for careful regulation of the fly's sleep and wake throughout the day.

One essential mechanism of this communication is neuromodulation. Neuromodulation is the altering of synaptic effectiveness of the neurons in a particular circuit [26]. Neuromodulators manipulate the ability of a neuron to respond to classical synaptic inputs, allowing for gradations of cellular responses [26]. This typically occurs via G‐protein‐coupled receptors (GPCRs) to alter neuronal function, often for long periods of time [27]. Neuropeptides make up a diverse subset of neuromodulators and have important roles in regulation of motivated behaviors [28]. Neuropeptides have been found to act in a wide array of functions in *Drosophila*, acting both locally within a neural circuit and more broadly throughout the body of the fly [27, 29, 30]. Understanding the role of neuropeptides in sleep regulation in *Drosophila* is an important step in understanding both the cellular mechanisms behind the regulation of sleep behavior and the principles of how neurons within a circuit interact more broadly.

One of the most widely expressed neuropeptides is short neuropeptide F, or sNPF [29]. The sNPF gene is a precursor for four distinct sNPF peptides [31], which bind to a G‐coupled protein receptor that is expressed throughout the body of adult fruit flies and has strong homology to the NPY Y_2_ receptor found in mammals [27, 32]. sNPF is expressed in a variety of neuron types, spanning the protocerebrum, pars intercerebralis, dorsal medial neurons, subesophageal ganglion, optic lobes, and mushroom bodies, suggesting that the neuropeptide may have many different roles in these varying regions [33]. sNPF is also expressed in the master clock of the fly brain, in two of the six LNds and four of the five s‐LNvs [34]. sNPF has been found to colocalize with several other neurotransmitters, including acetylcholine, GABA, and glutamate [33], as well as other molecules such as cryptochrome (CRY) in the LNds and s‐LNvs [35, 36], and additionally with the neuropeptide pigment‐dispersing factor (PDF) in the s‐LNvs [37, 38]. Furthermore, sNPF is expressed in the intrinsic Kenyon cells of the mushroom bodies [39], bilateral symmetrical neuropil structures that contain both sleep‐ and wake‐promoting neurons [2].

This broad expression of sNPF indicates that the neuropeptide likely plays more than one role in the *Drosophila* brain. Indeed, it has previously been found to play roles in insulin signaling [40], food intake and body size [41, 42], and, of interest to this study, sleep [43, 44, 45]. Studies of flies mutant for sNPF and studies using either thermogenetic or optogenetic activation of sNPF neurons have found a sleep‐promoting role for sNPF and the neurons that produce it [44, 45], but see Chen et al. [43]. In our current study, we utilized optogenetics, in which light‐responsive channels can be transgenically expressed in neurons of interest and then activated with light to produce electrical excitation of those neurons with precise timing [46, 47]. By expressing the red‐light sensitive channelrhodopsin Chrimson in sNPF neurons using the GAL4/UAS system, sNPF neurons can be activated with red‐light stimulation [48], which is less inherently disruptive to sleep and circadian rhythms. We recently found that this method of activation of sNPF neurons, for as little as 3 s, resulted in a persistent increase in sleep over a period of hours [44]. The same study also found that flies could be aroused from this sleep state, showing that the resting state induced by sNPF neuron activation was reversible like normal sleep, and was not an abnormal, irreversible state such as coma or locomotor dysfunction.

Because sNPF is expressed in a variety of brain regions, it remains unclear which populations of sNPF neurons are involved in this powerful sleep response. In order to address these questions, we have employed transgenes expressing the GAL4 inhibitor GAL80 [49] to selectively inhibit activation of subpopulations of sNPF‐positive neurons. By utilizing CRY‐GAL80, PDF‐GAL80, and MB‐GAL80 transgenes, Chrimson expression can be inhibited in CRY‐expressing clock neurons, the PDF‐expressing LNvs, and in the mushroom bodies, respectively. We have thus examined the roles of these neuron populations in the regulation of sleep by sNPF neurons.

## 2 Materials and Methods

### 2.1 Fly Lines and Maintenance

We used the following transgenic *Drosophila melanogaster* strains: w‐;sNPF‐GAL4/CyO (from Michael Rosbash Lab, Brandeis University, referred to here as sNPF‐GAL4), w‐;UAS‐CsChrimson (Bloomington *Drosophila* Stock Center, # BL55136, referred to here as UAS‐CHR), w‐; 40XUAS‐mCD8::GFP (BL32195), w‐.10XUAS‐mCD8::GFP; (BL32189, from Leslie Griffith lab, Brandeis University) w‐; MB‐GAL80 (from Leslie Griffith lab, Brandeis University, generated by Krashes et al. [50], referred to here as MB‐GAL80), w‐; CRY‐GAL80 (BD3F1); CRY‐GAL80 (2E3M)/TM6B (from Leslie Griffith Lab, Brandeis University, generated by 2004, referred to here as CRY‐GAL80), w‐; PDF‐GAL80/CyO; PDF‐GAL80^45x^ (Leslie Griffith Lab, Brandeis University, referred to here as PDF‐GAL80). All of these strains had been backcrossed with our control w^1118^ CS line (originally from the Leslie Griffith lab, Brandeis University, referred to here as w‐). We created a w‐; sNPF‐GAL4/CyO; UAS‐CHR/TM6B, Hu line, males of which were then crossed with w‐ to generate w‐; sNPF‐GAL4/+; UAS‐CsChrimson/+ progeny, henceforth referred to as sNPF > CHR flies, or to CRY‐GAL80, PDF‐GAL80, or MB‐GAL80 virgin females. Negative controls were created by crossing w‐ males with virgin females of the same GAL80 lines. We did not include controls containing UAS‐CsChrimson alone, as these were already included in our previous publication by Juneau et al. [44], and we found, as expected, that these flies did not show an increase in baseline sleep or in sleep in response to red light. We also did not include “No ATR” controls, as it has already been reliably demonstrated that effects on sleep of optogenetic activation by red‐light stimulation of CsChrimson are dependent on the presence of ATR [51, 52]. Flies were maintained in plastic vials or bottles (Genesee Scientific) containing fly food at 25°C, on a 12 h light/12 h dark (LD) cycle. Fly food consisted of 4 L distilled H_2_O, 400 g cornmeal (Quaker yellow), 24 g agar (*Drosophila* agar, 100 mesh), 72 g yeast (Red Star active dry yeast), 240 g dextrose, 120 g sucrose, 2 g methyl paraben (Josh's Frogs), and 40 mL of a mix of propionic acid (41.8%) and phosphoric acid (4.15%). Cornmeal, agar, and yeast were all from Genesee Scientific, and dextrose, sucrose, propionic acid, and phosphoric acid were all from Fisher Scientific. All incubators used for housing and experimentation were from Percival Scientific. All experimental crosses were set up on food of the type described above, which had been melted and combined with 1 mM all‐*trans*‐retinal (ATR; Toronto Research Chemicals, R240000, prepared as a 100 mM stock in ethanol). Crosses were raised in dark: dark (DD) conditions in a separate 25°C incubator. DD conditions were used in order to prevent unwanted optogenetic activation during development, because even moderate ambient white light can be sufficient to activate the Chrimson sensor. Progeny were collected 3–5 days prior to experimentation. We focused our analysis on female flies due to the high levels of sleep that had been previously observed in male flies [44]. Due to previously established differences in sleep patterns between virgin female and mated female flies, all females were mated prior to experimentation [53, 54, 55].

### 2.2 Sleep Experiments

**FIGURE 1:**
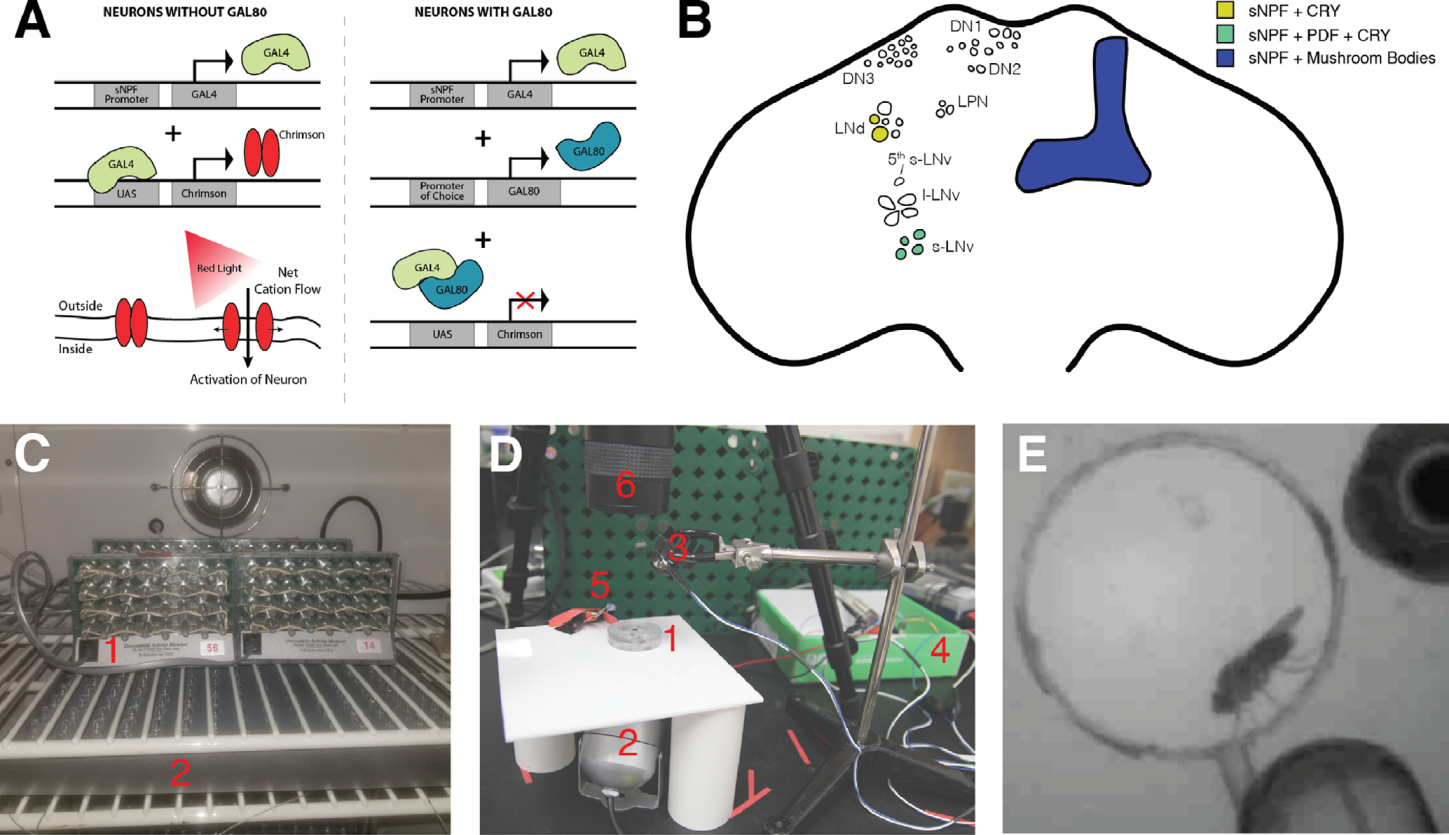
Methodological approaches used in this study. (A) Genetic approach used to test the roles of specific sNPF‐expressing neuronal populations in regulating sleep. On the left, in neurons that express GAL4 but not GAL80, GAL4 is active and can drive expression of a target gene (in our case, Chrimson). This allows those neurons to be activated by red light optogenetically. On the right, in neurons expressing both GAL4 and GAL80, GAL4 is inhibited and Chrimson cannot be expressed. (B) Schematic of the brain of the adult fruit fly *Drosophila melanogaster* , indicating selected neurons and brain regions where sNPF is expressed. Color codes indicate locations that are targeted by the sNPF‐GAL4 driver and GAL80 repressors. Clear cells are non‐sNPF‐expressing clock cells. Additional sNPF‐positive neurons outside of the clock network and mushroom bodies exist but are not depicted. Note that in the actual fly, expression is bilateral, but for simplicity this diagram depicts clock neurons on the left and mushroom bodies on the right. (C) Sleep experiment apparatus: Flies were placed in individual tubes in DAM2 monitors (1) inside of an incubator and were illuminated from below with a 125‐LED red light grid (2). (D) Acute experiment apparatus: Flies were added to a chamber (1) placed on a light diffusing board over an infrared light source (2). The red LED light (3) for stimulation was controlled by an Arduino board (4). An infrared indicator light (5) was controlled by the same timer to indicate when the red light was turned on. This was necessary because a camera (6) equipped with a long‐pass infrared filter was used to capture video. (E) A sample screenshot from a video recording, showing the size of the chamber relative to the fly. The infrared indicator light can be seen in the bottom right.

Flies were loaded into *Drosophila* Activity Monitors (DAMs) (Trikinetics) and placed in a dark incubator directly over a red‐light LED grid at 25°C (Figure 1C). Circadian Time 0 (CT 0) was set as 9 a.m. Activity of the flies was monitored for 3 days: a baseline day, a stimulation day, and a recovery day. On the stimulation day, flies were stimulated using a red‐light LED grid with peak emission at 630 nm (HQRP) for either 15 min or 1 h at CT 7 or 8. Data were collected using DAMSystem3 software (Trikinetics), processed using DAMFileScan (Trikinetics), and further analyzed to calculate sleep metrics using Sleep and Circadian Analysis MATLAB Program (SCAMP) [56] in MATLAB (Mathworks). To analyze the change in sleep between the baseline and stimulation days, sleep per 30 min for each fly was calculated and each 30‐min bin on the baseline day was subtracted from the same bin on the stimulation day.

### 2.3 Acute Activity Level Experiments

Flies were aspirated individually into a courting chamber with a radius of 0.7 cm and a depth of 0.3 cm. The chamber was placed on top of a light‐diffusing panel over an infrared light source. A Sony CCD/R XC‐E150 camera with a M6Z1212‐3S Manual Zoom lens (Computar) attached in tandem via a VM‐100 extension tube (Computar) and equipped with an 850 nm Near‐IR Longpass M55 filter (MidOpt) was positioned on a tripod above the chamber. Video data was recorded to a MacBookPro using EasyCap Viewer software. After the fly was added to the chamber, we initiated an Arduino program using an Arduino Genuino board (Figure 1). This program included a 30‐s baseline period, before turning on a red LED light for 5 s. The red LED was positioned above the well, such that the intensity of the light on the fly was an average of 4.424 mW. Video recording then continued for an additional 2.5 min. Videos were hand scored by a blind observer for time spent walking, grooming, and resting during each 30‐s bin of the recording (excluding the 5‐s stimulation period).

### 2.4 Immunostaining and Confocal Imaging

For experiments run at Skidmore College, sample flies were dissected in cold 0 mM Ca^2+^ *Drosophila* adult hemolymph‐like saline (AHL) [57]. Brains were then fixed in Zamboni's fixative (American Master Tech, FXZAMLT) for at least 30 min at room temperature (RT), washed quickly in phosphate‐buffered saline (PBS; Sigma, P3813), and then rinsed in PBS two more times for at least 10–20 min at RT. Before primary antibody treatments, brains were washed 3x for 10–20 min at RT in PBS containing 0.3% Triton‐X (Integra, T756.30.30) (PBTx), and were then incubated in blocking buffer (5% normal goat serum [NGS; Sigma, G6767] in PBTx) for at least 1 h. Brains were incubated in primary antibodies for four hours at room temperature or overnight at 4°C. All primary antibodies were diluted in blocking buffer. Samples were washed 3x in PBTx for 10–20 min at RT and then incubated in secondary antibodies for 4 h at room temperature or overnight at 4°C. All secondary antibodies were diluted in blocking buffer. Brains were then washed in PBTx and whole‐mounted in Slow Fade Diamond Antifade Mountant (Invitrogen S36936). Slides were imaged using an Olympus FV1200 confocal laser scanning microscope using either ×20 air or ×40 oil objectives, using Fluoview FV1000 software. Laser power was kept constant within imaging sessions. Photomultiplier tube high voltage (HV) levels, gain, and offset were adjusted brain by brain to optimize brightness while avoiding oversaturation and reducing background noise. The aspect ratio was set to 1600 × 1200 pixels and the dwell time was 10 μs/pixel. Z‐stack images were made up of 80–95 slices with a step size of 1.00–1.50 μm.

For experiments run at West Virginia University, dissections were performed in *Drosophila* saline [58]. For Timeless labeling, dissections were specifically performed at ZT 20. Samples were fixed in 4% paraformaldehyde (Electron Microscopy Services, 15,710) for 30 min to 1 h at 4°C. Samples were then washed 4x in PBST (PBS; Sigma, P3813) with 0.5% Triton X‐100 (Sigma, 93443) and blocked in 4% bovine serum albumin (BSA; Jackson Immunoresearch Laboratories, 001‐000‐173) in PBST and 50 mM sodium azide (Sigma, s8032). Samples being labeled for PDF or Timeless were instead treated with a blocking buffer consisting of 3% normal goat serum (Invitrogen, PCN5000) in PBST and 50 mM sodium azide. Primary antibodies (see Table 1 for antibody details) were applied and incubated for 48 h at 4°C with agitation. Samples were then washed 4x in PBST and blocked as described above. Secondary antibodies were then applied and incubated for 48 h at 4°C with agitation. Samples were then washed 2X in PBST and 2X in PBS and then cleared in a glycerol (Carolina Biological, 865530) series (40%, 60%, and 80%) for 10 min each. Samples were then mounted in Everbrite (Biotium, 23001) on well slides. Images were captured using an Olympus FV1000 confocal with a 40x UPlanFL‐N silicone oil immersion lens, using Fluoview FV1000 software. Laser power was kept constant within imaging sessions. Gain and offset levels were adjusted brain by brain to avoid oversaturation and reduce background noise.

**TABLE 1:** Antibodies used for immunostaining experiments.

Antibody	Source	Dilution	Catalog number
Skidmore college			
Mouse anti‐nc82	DSHB	1:20	AB_2314866
Rabbit anti‐GFP	Life technologies	1:1000	A11122
Goat anti‐Rabbit AlexaFluor 488	Life technologies	1:800	A11034
Goat anti‐Mouse AlexaFluor 568	Life technologies	1:400	A11031
West virginia university			
Chicken anti‐GFP	Abcam	1:1000	AB 13970
Rat anti‐NCAD	DSHB	1:500	DN‐EX #8
Rat anti‐Timeless	Gift from Michael Rosbash	1:200	
Mouse anti‐PDF	Gift from Jan Veenstra	1:200	
Mouse anti‐nc82	DSHB	1:100	AB_2314866
Donkey anti‐Chicken AlexaFluor 488	Jackson ImmunoResearch Laboratories	1:1000	#703–545‐155
Donkey anti‐Mouse AlexaFluor 546	Life Technologies	1:1000	A10036
Donkey anti‐Rat AlexaFluor 647	Abcam	1:1000	AB_2813835

Data (.oib format) from confocal imaging were processed using ImageJ with a Bio‐Formats plug‐in.

### 2.5 SCOPE Data Mining

The aging adult fly brain dataset from Davie et al. [59] was analyzed by searching for sNPF, CRY, and TIM expression across all cell samples. These data were CPM‐normalized and exported to an Excel sheet, where the results were further filtered to include only cells from flies ages 6–9 days old, separated by sex. Data were then filtered based on whether cells expressed one or multiple of the genes of interest. Based on the total number of sNPF‐positive cells, fractions of that total were calculated for cells that co‐expressed CRY or TIM as well.

### 2.6 Statistical Analysis

For sleep experiments, subtraction data were analyzed statistically using mixed‐model analysis of variances (ANOVAs) with genotype as the between‐subject factor and time bin as the within‐subject factor. Tukey's HSD tests were used for post hoc analysis using a threshold for significance of *p* < 0.05. From that analysis, we have reported two separate types of comparisons: First, a comparison across genotypes for a given bin (e.g., sNPF/CHR had a significantly greater increase in sleep than sNPF/CHR + CRY‐GAL80 in the third bin after 15 mins of red‐light stimulation—these are the statistical results shown in the figures). Second, a comparison across bins within a given genotype, specifically reporting differences during and post‐stimulation relative to the sleep subtraction value immediately preceding stimulation (e.g., changes in sleep relative to the baseline day were significantly greater in sNPF/CHR flies both during and for several bins following 15 mins of red‐light stimulation than they were immediately preceding stimulation). Post‐activation sleep/wake architecture metrics were analyzed over four 6‐h bins using SCAMP [56], where the first bin began on the first minute of optogenetic stimulation. Mixed‐model ANOVAs were then run on each analysis type with genotype as the between‐subject factor and time bin as the within‐subject factor. When significant Genotype × Bin interactions were found, Tukey's HSD tests were used for post hoc analysis using a threshold for significance of *p* < 0.05. Pdoze analysis requires that flies be awake and then fall asleep. However, many flies, especially in the sNPF > CHR groups, stayed asleep for the entirety of some 6‐h bins following optogenetic activation. Thus, due to missing data points, Pdoze could not be analyzed statistically, although the results are shown graphically. All statistical analysis was carried out using JMP Pro (versions 13, 14, and 15). The data that support the findings of this study are available from the corresponding author upon reasonable request.

## 3 Results

### 3.1 Cryptochrome‐Positive but PDF‐Negative Clock Neurons Contribute to Sleep Induction due to sNPF Neuron Activation

**FIGURE 2:**
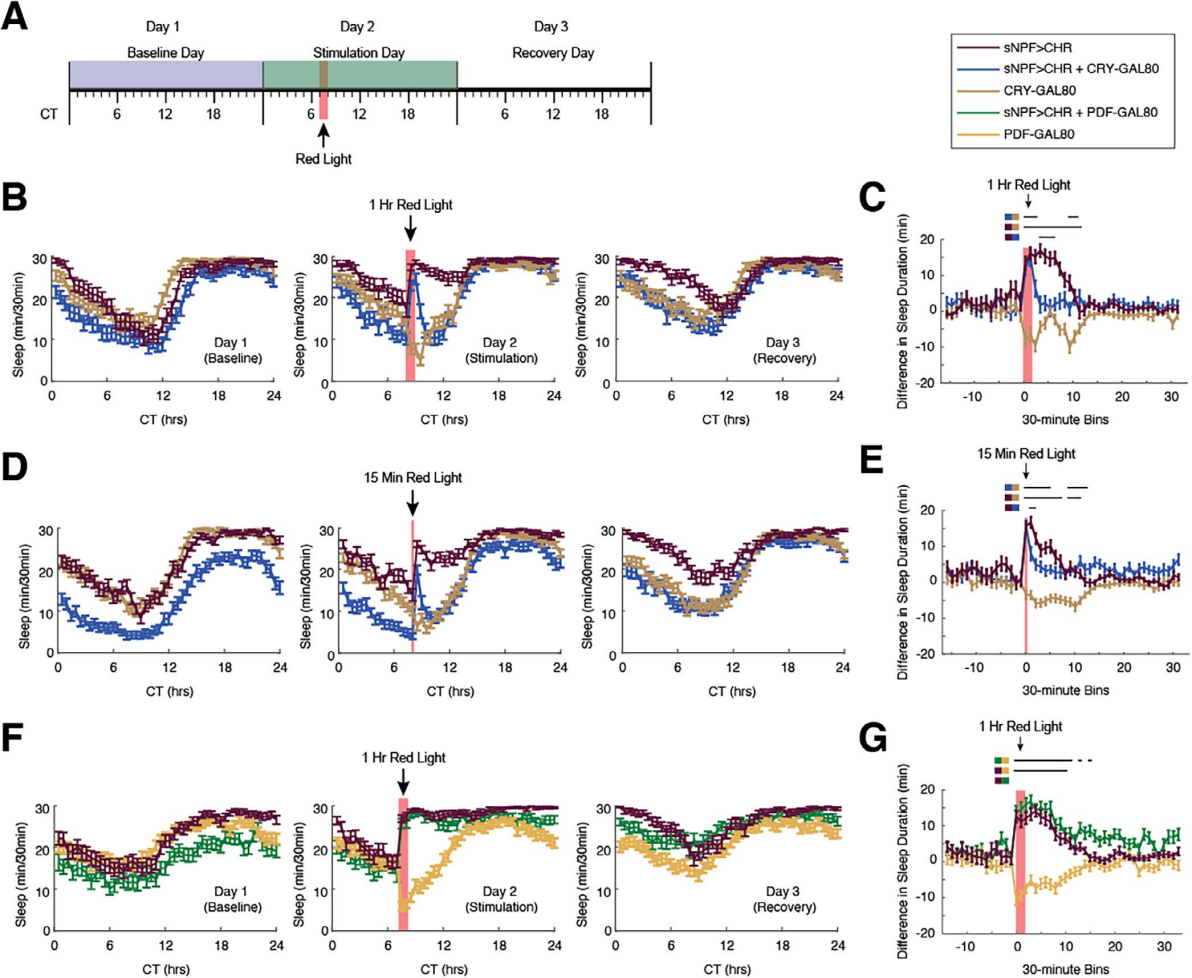
sNPF neurons that express Cryptochrome but not PDF are required for long‐lasting sleep induction. (A) Timeline of sleep experiments. Red light was applied on the second day. To calculate sleep induction (see panels C, E, and G), sleep during the baseline day was subtracted from sleep during the stimulation day. (B, C) CRY‐GAL80, 1‐h stimulation. (B) Daily amounts of sleep in 30‐min bins across the three recording days, during which 1 h of red light was applied on Day 2. (C) Sleep subtraction plot for 1 h of red‐light stimulation. (D, E) CRY‐GAL80, 15‐min stimulation (D) Similar to (B), but for data in which red light was applied for 15 min. (E) Sleep subtraction for the 15‐min stimulation data. As can be seen in both (C) and (E), sleep induction persisted longer when all sNPF neurons were activated, as compared to when CRY‐positive neurons were excluded from being activated. (F, G) PDF‐GAL80, 1‐h stimulation. Unlike results with CRY‐GAL80, inclusion of PDF‐GAL80 did not reduce the longevity of the sleep induction effect. All graphs depict means ± SEM. Lines above the subtraction plots point out bins where the two groups indicated by the colored squares were significantly different from each other. Significant differences were calculated using Tukey post hoc tests. *N'*s for the 1‐h CRY data were 55, 45, and 61 for sNPF > CHR, sNPF > CHR + CRY‐GAL80, and CRY‐GAL80, respectively. *N*'s for the 15‐min CRY data were 66, 58, and 95 for sNPF > CHR, sNPF > CHR + CRY‐GAL80, and CRY‐GAL80, respectively. *N*'s for the 1‐h PDF data were 59, 49, and 72 for sNPF > CHR, sNPF > CHR + PDF‐GAL80, and PDF‐GAL80, respectively. All data were from female flies.

To examine the role of clock neurons in the sleep response to sNPF neuron activation, flies with sNPF‐GAL4 and UAS‐Chrimson transgenes (sNPF > CHR), CRY‐GAL80 Alone, or all three transgenes (sNPF > CHR + CRY‐GAL80) were exposed to red‐light stimulation at CT8 (Figure 2A). This time was chosen because it is during the subjective daytime period when flies normally exhibit a period of high activity, thus allowing for a sleep response to be fully seen. One hour of stimulation induced a prolonged increase in sleep in sNPF > CHR flies that was significantly greater than in controls both during the red‐light stimulus and for 5 h after the stimulation had ceased (Figure 2B,C), corroborating our previous results [44]. However, when Chrimson expression was excluded from cryptochrome‐positive neurons using CRY‐GAL80, the duration of this effect was substantially decreased (Figure 2B,C). An ANOVA based on the subtraction data in Figure 2C found a significant interaction between genotype and time bin (*F*(94, 7426) = 8.51, *p* < 0.0001). Tukey post hoc testing showed that sleep in sNPF > CHR flies was increased significantly more than in CRY‐GAL80 controls for the two 30‐min during the red‐light stimulation and for 10 subsequent bins. In contrast, the increase in sleep seen in sNPF > CHR + CRY‐GAL80 flies was only significantly greater than in CRY‐GAL80 controls for the two bins during the stimulation and one subsequent bin (post‐stimulation bin 1) before returning to control levels for several hours. The two groups were later significantly different again during post‐stimulation bins 11–12, although this was mainly due to a decrease in sleep in the controls. During post‐stimulation bins 4–7, sleep was still increased in sNPF > CHR flies, but significantly less so in sNPF > CHR + CRY‐GAL80 flies. On the recovery day, flies in which all sNPF neurons were activated continued to sleep somewhat more than control flies and sNPF > CHR + CRY‐GAL80 flies during the subjective daytime period. However, the timing of sleep patterns on the recovery day was qualitatively similar across all groups, suggesting that activating sNPF neurons did not influence circadian rhythms.

Analyses were also run comparing baseline, stimulation, and post‐stimulation sleep levels within each fly genotype, based on the same Tukey post hoc tests from the main ANOVA for the 1‐h stimulation CRY‐GAL80 experiment. The half‐hour bin immediately prior to optogenetic stimulation was used as a baseline value in these comparisons since all baseline bins (bins 1–16) were statistically similar within genotypes, and this bin directly preceded the stimulation bins. Comparing sleep levels between the pre‐stimulation bin and subsequent bins in sNPF > CHR flies, both stimulation bins as well as post‐stimulation bins 1–8 had significantly higher levels of sleep compared to the pre‐stimulation bin. However, sNPF > CHR + CRY‐GAL80 flies only had increased sleep compared with the pre‐stimulation bin during the period of stimulation but not afterward. CRY‐GAL80 control flies showed significantly lower levels of sleep in post‐stimulation bins 1 and 8, but otherwise were unaffected by the stimulation.

We next carried out more fine‐grained analysis of how optogenetic activation of sNPF neurons affected sleep/wake parameters over four successive 6‐h bins starting at the onset of red‐light stimulation (Table S1 and Figure S1). To assess sleep architecture, we analyzed total sleep, sleep episode number, and mean sleep episode duration. We also assessed flies' activity per minute awake, as an indicator of whether flies had any locomotor issues or if they were generally hypo/hyperactive. Finally, we analyzed probabilistic measures of sleep pressure (Pdoze) and sleep depth (Pwake) [60]. In the 1‐h CRY‐GAL80 experiment (Figure S1A), we found that activation of all sNPF neurons strongly increased total sleep time during the first 6‐h period post‐activation, as well as the third and fourth 6‐h periods, spanning from 12 to 24 h post‐activation. The inclusion of the CRY‐GAL80 transgene prevented all of these long‐term increases in total sleep. Activation of all sNPF neurons caused a general decrease in the number of sleep episodes across the full 24‐h post‐activation period, and a strong increase in sleep episode duration during the first 6‐h period. Again, inclusion of the CRY‐GAL80 transgene counteracted these effects, especially the effect on episode duration. This suggests that the increased sleep time was due more to flies staying asleep longer rather than going to sleep more often. A decrease in activity while awake could indicate that activation of sNPF neurons was causing apparent sleep effects by generally inhibiting movement. However, we found that activity per minute awake actually increased in sNPF > CHR flies relative to controls during the first 6‐h period following activation. This suggests instead that sNPF neuron activation is in fact changing flies' sleep drive. Analysis of Pdoze and Pwake corroborated the other sleep/wake metrics. Pdoze could not be analyzed statistically, because many sNPF > CHR flies spent whole 6‐h periods asleep, resulting in missing data points. The qualitative trend was for sNPF > CHR flies to have increased Pdoze, especially during the first 6‐h period post‐activation, but also during hours 12–24. But the more striking result was that activation of all sNPF neurons dramatically lowered Pwake, reaching significance in the 0–6‐h and 18–24‐h periods post‐activation. Flies with the CRY‐GAL80 transgene did not show these changes in Pwake. Overall, these analyses are supportive of a primary role of cryptochrome‐positive sNPF neurons in promoting sleep depth, with additional smaller effects on sleep pressure.

We also examined whether a shorter, 15‐min period of optogenetic stimulation would also be sensitive to the removal of cryptochrome‐expressing neurons from the overall sNPF‐GAL4 population. We found very similar results with 15 min of stimulation as with 60 min, in which sNPF > CHR and sNPF > CHR + CRY‐GAL80 flies showed a similar initial spike in sleep, but the increased sleep in sNPF > CHR flies persisted longer than it did in sNPF > CHR + CRY‐GAL80 flies (Figure 2D,E). ANOVA results from the subtraction analysis found a significant interaction between genotype and time bin (*F*(94, 10,152) = 6.81, *p* < 0.0001). Tukey post hoc testing showed that sNPF > CHR flies differed significantly from CRY‐GAL80 x w‐ female flies during the 30‐min bin when red‐light stimulation was given and for the following seven 30‐min periods, as well as post‐stimulation bins 10–11. In contrast, sNPF > CHR + CRY‐GAL80 flies only had a significantly different sleep response than CRY‐GAL80 flies during the bin when the red‐light stimulation occurred and for five subsequent bins, as well as post‐stimulation bins 10–12. The sleep induction in sNPF > CHR + CRY‐GAL80 flies was also significantly lower than in sNPF > CHR flies during the bin immediately following stimulation. As in the 1‐h experiment, sNPF > CHR flies continued to have elevated sleep compared with the other two groups during the subjective daytime period on the recovery day, but the timing of that sleep was not altered.

Comparing how sleep subtraction data changed within each genotype relative to pre‐stimulus levels for the 15‐min stimulus study, the sNPF > CHR flies exhibited significantly higher levels of sleep during the stimulation bin and the five subsequent bins. In contrast, sNPF > CHR + CRY‐GAL80 flies only showed significantly higher levels of sleep during the stimulation bin, but not afterward. CRY‐GAL80 control flies did not have any significant changes to sleep when compared to the bin preceding the stimulus. Together, these results suggested that Cryptochrome‐positive clock neurons are important mediators of the later phase of the sleep response due to sNPF neuron activation. However, the initial sleep induction appeared to be independent of Cryptochrome neurons.

Analysis of sleep/wake architecture (Table S1 and Figure S1) found very similar effects of 15‐min sNPF neuron activation to those found for 1‐h activation on total sleep, sleep bout length, activity while awake, Pdoze, and Pwake. The only notable difference was that sNPF > CHR + CRY‐GAL80 flies had more fragmented sleep in this experiment, as evidenced by their high numbers of short sleep episodes (Figure S1B). But overall, analysis of the 15‐min activation data supported the conclusion that activation of Cryptochrome‐positive sNPF neurons promotes sleep depth.

Cryptochrome is expressed in several clusters of clock neurons, including the l‐LNvs and s‐LNvs [35], both of which also express the neuropeptide PDF [37, 38]. The s‐LNvs have been shown to express sNPF, whereas the l‐LNvs do not [34]. To determine if these neurons that co‐express sNPF, PDF, and Cryptochrome are the relevant neurons generating the long‐lasting sleep increase following activation of all sNPF neurons, we utilized PDF‐GAL80 to exclude Chrimson expression from PDF neurons. In contrast to the results using CRY‐GAL80, sNPF > CHR + PDF‐GAL80 flies showed a long‐lasting increase in sleep during and following red‐light stimulation, that was more prolonged than in sNPF > CHR flies (Figure 2F,G). ANOVA results from the subtraction analysis found a significant interaction between genotype and time bin (*F*(94, 8319) = 9.753, *p* < 0.0001). Tukey post hoc testing showed that both sNPF > CHR and sNPF > CHR + PDF‐GAL80 flies had significantly different sleep responses compared with PDF‐GAL80 controls for the hour of stimulation and for 4.5 h afterward (post‐stimulation bins 1–9). The sNPF > CHR + PDF‐GAL80 group also differed from controls during post‐stimulation bins 10, 12, and 14. On the recovery day, flies in which all sNPF neurons were activated continued to sleep more than control flies during the subjective daytime period, with sNPF > CHR + PDF‐GAL80 flies showing an intermediate level of sleep. As in the CRY‐GAL80 experiments, the timing of sleep patterns on the recovery day was qualitatively similar across all groups.

When comparing sleep subtraction data during and after the red‐light stimulus with sleep during the pre‐stimulation 30‐min bin for each genotype, flies in the sNPF > CHR group showed significantly higher levels of sleep during both stimulation bins and the six subsequent bins. Similarly, the sNPF > CHR + PDF‐GAL80 flies exhibited significantly higher levels of sleep during the two stimulation bins and eight subsequent bins. PDF‐GAL80 control flies showed significantly lower levels of sleep during the two bins of stimulation compared to the pre‐stimulus bin, and no changes during the post‐stimulation period. Combined with our CRY‐GAL80 results, these data suggest that PDF‐positive clock neurons such as the s‐LNvs are not the critical drivers of sleep due to sNPF neuron activation, but that other Cryptochrome‐positive clock neurons are.

Analysis of sleep/wake architecture (Table S1 and Figure S1) found that activation of all sNPF neurons had similar effects compared with the 1‐h and 15‐min CRY‐GAL80 experiments described above on total sleep, sleep episode frequency, mean sleep episode duration, activity while awake, Pdoze, and Pwake (Figure S1C). However, in this case, the inclusion of the PDF‐GAL80 transgene did not prevent those effects on sleep, supporting the conclusion that PDF‐positive neurons are not critical for regulation of sleep patterns by sNPF neuron activation.

### 3.2 Roles of Cryptochrome‐ and PDF‐Positive Clock Neurons in Acute Behavioral Responses to Brief sNPF Neuron Activation

**FIGURE 3:**
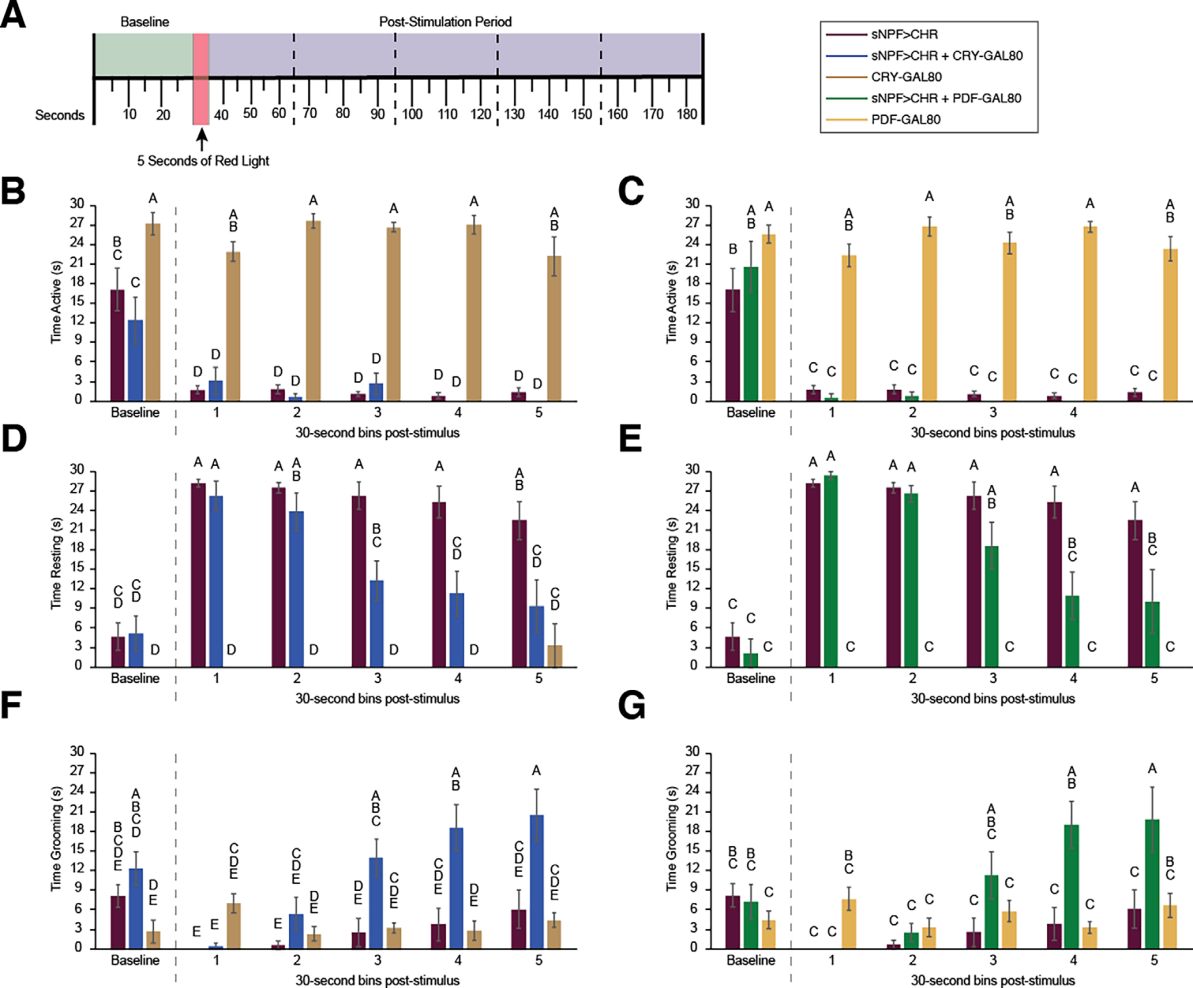
Roles of Cryptochrome‐ and PDF‐positive neurons in short‐term behavioral responses to activation of sNPF neurons. (A) Timeline for acute stimulation experiments. Video was recorded during the 30‐s baseline period, the 5‐s red light stimulation period, and 2.5 min (5 × 30‐s bins) following stimulation. Videos were scored for time spent active (but not grooming), resting (completely still), and grooming. Panels (B), (D), and (F) show results from Cryptochrome (CRY)‐related fly lines, and panels (C), (E), and (G) show results from pigment dispersing factor (PDF)‐related fly lines. The same data from the sNPF > CHR line are plotted on both sets of graphs. Letters above bars represent significance groups—any groups that do not share a letter are significantly different from each other. Statistical significance was calculated by Tukey post hoc tests. All graphs depict means ± SEM. *N*'s were 11 for sNPF > CHR, nine for sNPF > CHR + CRY‐GAL80, nine for CRY‐GAL80, seven for sNPF > CHR + PDF‐GAL80, and seven for PDF‐GAL80. All data were from female flies.

Although activity monitors are effective for obtaining data about prolonged sleep and activity patterns from large groups of flies, they do not provide information about the actual behaviors engaged in by the flies in response to optogenetic stimulation. Therefore, we carried out acute stimulation experiments in which we videotaped individual flies and scored time spent actively walking, resting, and grooming in response to 5 s of red‐light stimulation (Figure 3A). For CRY‐GAL80 groups, we found significant interactions between genotype and time bin for time spent actively walking (*F*(10, 130) = 3.975, *p* < 0.0001) (Figure 3B), time spent resting (*F*(10, 130) = 7.934, *p* < 0.0001) (Figure 3D), and time spent grooming (*F*(10, 130) = 6.096, *p* < 0.0001) (Figure 3F). Tukey post hoc analysis showed that, during the baseline, both sNPF > CHR and sNPF > CHR + CRY‐GAL80 spent less time actively walking than did CRY‐GAL80 controls, although time spent resting or grooming did not differ among groups. Red‐light stimulation resulted in a significant reduction in activity in both experimental groups that persisted for the 2.5‐min duration of the video recording (see Video S2), whereas the controls did not show any change in activity due to red‐light stimulation (Figure 3B). Immediately following the stimulus, sNPF > CHR and sNPF > CHR + CRY‐GAL80 flies both had significantly increased resting time. However, the two groups began to diverge over time, as the sNPF > CHR group continued to spend virtually all of their time resting, whereas the sNPF > CHR + CRY‐GAL80 group's resting level declined through the recording (Figure 3D) and grooming levels gradually increased (Figure 3F).

For PDF‐GAL80 groups, significant interactions between genotype and time bin were found for time spent walking (*F*(10, 110) = 6.719, *p* < 0.0001), resting (*F*(10, 110) = 9.691, *p* < 0.0001), and grooming (*F*(10, 110) = 5.898, *p* < 0.0001). Overall, results were very similar to those observed with CRY‐GAL80. Optogenetic stimulation of sNPF > CHR + PDF‐GAL80 flies significantly reduced their activity throughout the 2.5 min of the video recording (Figure 3C). Initially, they had the strongest increase in resting behavior, but this decreased toward the end of the recording (Figure 3E) and was replaced by grooming behavior (Figure 3G).

Our video‐based analysis also showed that flies in the sNPF > CHR, sNPF > CHR + CRY‐GAL80, and sNPF > CHR + PDF‐GAL80 groups all exhibited a period of uncoordinated movement during the red‐light stimulation, in which they would fall over and exhibit twitching of limbs with little or no coordinated movements such as walking or grooming. Soon after the end of the stimulus, they would return to a standing position (see Video S1). This initial reaction did not differ in any obvious way between flies in which all sNPF neurons were activated (sNPF > CHR) and flies in which either Cryptochrome‐ or PDF‐expressing neurons were excluded from being activated. Control flies (CRY‐GAL80 Alone and PDF‐GAL80 Alone) did not show this acute reaction to the red‐light stimulus. To ensure that this uncoordinated movement was not responsible for the decrease in movement following longer periods of red‐light stimulation used in our sleep experiments, we ran additional tests using a 15‐min period of red‐light stimulation. Uncoordinated movement was most pronounced during the first 10 s of stimulation, but it did continue intermittently through the 15 min of stimulation. Notably, as soon as optogenetic stimulation ceased, this uncoordinated behavior ended immediately, and flies were able to begin engaging in walking and grooming behavior (see Video S3). This was true in sNPF > CHR as well as sNPF > CHR + CRY‐GAL80 flies (see Video S4). Thus, these results, combined with our previous arousal analysis [44], make it highly unlikely that the sleep observed during the hours after optogenetic stimulation is due to immobilization.

### 3.3 Assessment of the Effects of CRY‐GAL80 and PDF‐GAL80 on sNPF‐GAL4‐Mediated Chrimson Expression

**FIGURE 4:**
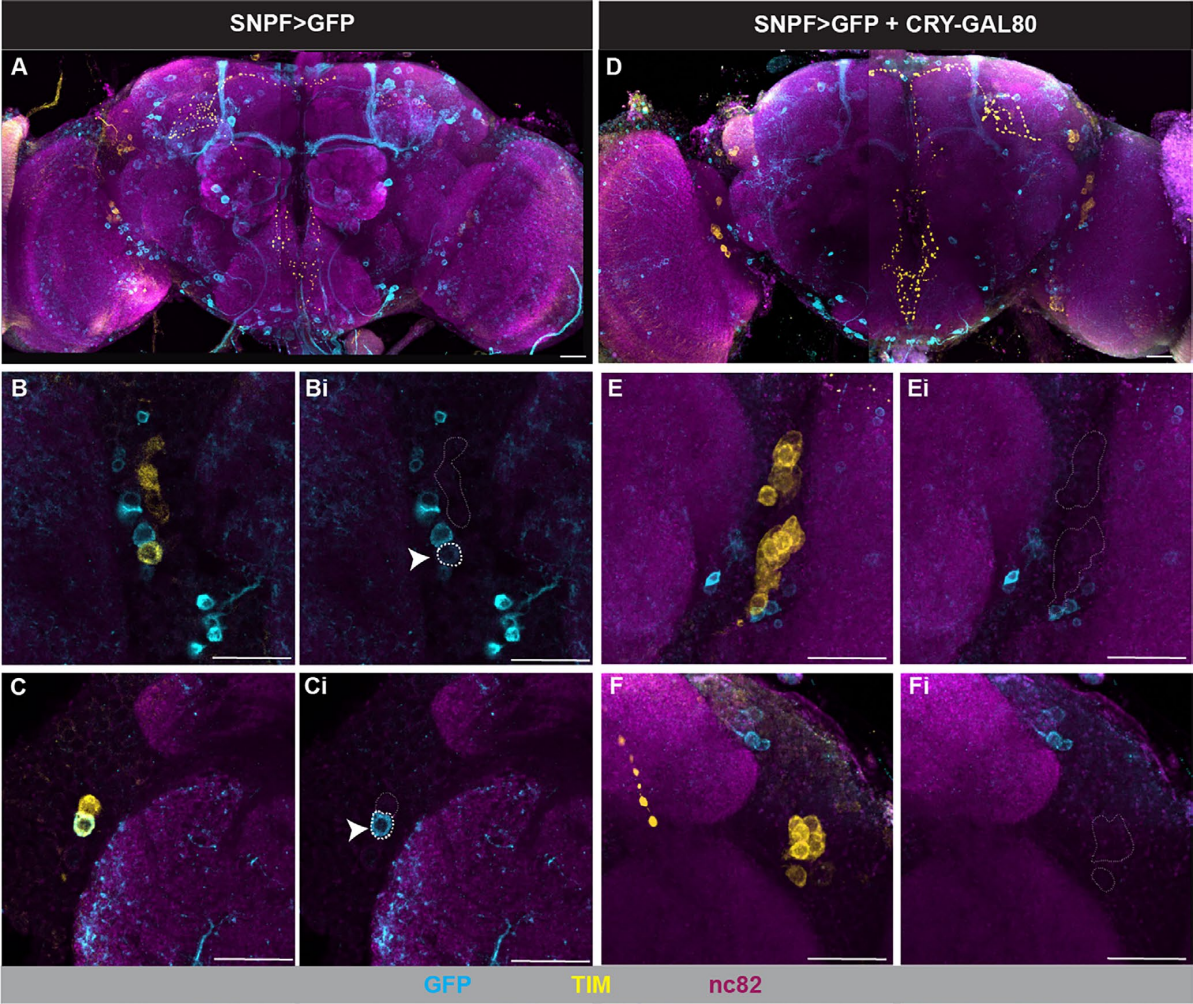
sNPF‐GAL4‐mediated expression of GFP in the absence and presence of CRY‐GAL80. Anti‐GFP immunostaining (cyan) detected the presence of mCD8::GFP driven by sNPF‐GAL4. Brains were also stained against Timeless (TIM; yellow) to detect clock neurons and nc82 (magenta) as an anatomical background. White dotted lines indicate cells expressing TIM and white arrows indicate overlap with GFP expression. (A) Full brain image in the absence of CRY‐GAL80. (B, Bi) Single slice images showing TIM expression in LNv cell bodies, with co‐staining in (B) and only anti‐GFP staining in Bi. (C, Ci) Single slice images showing TIM expression in LNd cell bodies, with co‐staining in (C) and only anti‐GFP staining in (Ci). (D) Full brain image of GFP expression in the presence of CRY‐GAL80. (E, Ei) Max projections of LNv cell bodies, with co‐staining in (E) and anti‐GFP staining only in (Ei). (F, Fi) Max projections of LNd cell bodies, with co‐staining in (F) and anti‐GFP staining only in (Fi). All scale bars represent 20 μm.

We next performed immunostaining and confocal imaging experiments to confirm that the CRY‐GAL80 and PDF‐GAL80 transgenes were in fact able to repress expression of Chrimson driven by sNPF‐GAL4. We dissected the brain and ventral nerve cord (VNC) from adult flies and immunostained against GFP, which allowed us to detect the mVenus tag on the CsChrimson protein [48]. Chrimson expression was consistently observed in four clusters of Kenyon cells within the calyx of the mushroom body (MB), with prominent projections into various MB lobes (Figure 4A and Video S5), as previously described [39]. Expression of Chrimson in the MB was still evident despite the addition of CRY‐GAL80 (Figure S1C) or PDF‐GAL80 (Figure S1D). We also co‐stained against Timeless (TIM) in order to visualize clock neuron populations, including the dorsolateral neurons (LNds) and small and large ventrolateral neurons (s‐LNvs and l‐LNvs, respectively), or against pigment dispersing factor (PDF) in order to visualize the s‐LNvs and l‐LNvs specifically. For TIM labeling, dissections were specifically performed at ZT20. All other dissections were performed during the daytime.

In sNPF > CHR flies, in which Chrimson should be expressed in all sNPF‐GAL4 neurons, we found that Chrimson expression overlapped with a few of the TIM‐expressing LNds (Figure S2A, and Video S5). However, this was only observed rarely. Specifically, out of 5 imaged brains, we saw overlap in just two LNds in one brain (Figure S2A). Surprisingly, we did not observe overlap of Chrimson with any of the TIM‐expressing s‐LNvs (Figure S2A) where sNPF had been detected previously [34]. Co‐staining against PDF found the same lack of overlap between Chrimson and the s‐LNvs (Figure S2B). In sNPF > CHR + CRY‐GAL80 flies, we never observed overlap between Chrimson and TIM‐positive LNds (Figures S2C, five imaged brains). In sNPF > CHR + PDF‐GAL80 flies, we also never observed overlap between Chrimson and PDF‐expressing LNvs (Figure S2D, three imaged brains), although this result was difficult to interpret since we did not observe overlap in the absence of PDF‐GAL80 either. To address this, we crossed our PDF‐GAL80 line with w.UAS‐mCD8::GFP;PDF‐GAL4/CyO, and found that PDF‐GAL80 was able to completely repress GAL4‐mediated expression of GFP in both the l‐LNvs and s‐LNvs (Figure S4. These results were consistent across *n* = 3 controls without PDF‐GAL80 and *n* = 4 experimental animals containing PDF‐GAL80). This strongly suggests that PDF‐GAL80 in our experiments had the ability to eliminate Chrimson expression in LNv neurons.

We also examined whether the addition of CRY‐GAL80 or PDF‐GAL80 affected expression of Chrimson driven by sNPF‐GAL4 within the VNC (Figure S3, Videos S6 and S7). We observed many Chrimson‐positive cell bodies and projections within the VNC, and a few TIM‐positive and PDF‐positive cells at the distal end of the VNC [61], as well as TIM‐positive puncta along projection tracks primarily near the midline of the VNC. However, there was never any overlap of Chrimson with either TIM (Figures S3A, four imaged VNCs) or PDF within the VNC (Figure S3B, four imaged VNCs). The addition of either CRY‐GAL80 (Figure S3C, four imaged VNCs) or PDF‐GAL80 (Figure S3D, four imaged VNCs) did not affect VNC expression of Chrimson in an obvious way. Overall, our imaging of the VNC suggested that CRY‐GAL80 was not likely to be having an effect on sleep promotion by repressing Chrimson expression within the VNC.

### 3.4 Assessment of the Effects of CRY‐GAL80 and PDF‐GAL80 on sNPF‐GAL4‐Mediated GFP Expression

The low amount of sNPF‐GAL4‐driven expression of Chrimson in the LNds and s‐LNvs could have been due to weak staining for the mVenus tag on the Chrimson protein using anti‐GFP antibodies. Therefore, we immunostained for GFP and TIM in sNPF‐GAL4 > 40xUAS‐mCD8::GFP flies. In these GFP‐expressing flies, we did see slightly greater overlap of GFP with TIM in the LNds and s‐LNvs than we had observed for Chrimson‐mVenus (Figure 4 and Table 2), but still not the two LNds and approximately 4 s‐LNvs per hemisphere predicted by the literature [34]. Additionally, the overlap we observed was primarily seen in male flies, but not in females (Table 2). As expected, we never saw overlap of GFP and TIM in the l‐LNvs. In sNPF > GFP + CRY‐GAL80 flies, we did not observe any overlap of GFP and TIM in the LNds or s‐LNvs in male flies (Figure 4 and Table 3), suggesting that CRY‐GAL80 was effective at repressing GAL4‐mediated gene expression in CRY‐expressing clock cells.

**TABLE 2:** Quantification of overlap of GFP and Timeless (TIM) staining in sNPF‐GAL4+40xUAS‐mCD8::GFP flies.

Cell type	Animals	Countable hemispheres	Total TIM+ cells	Cells with GFP overlap
Male				
LNds	5	8	45	2
s‐LNvs	4	7	29	3
l‐LNvs	4	7	25	0
Female				
LNds	3	5	30	0
s‐LNvs	2	4	20	0
l‐LNvs	3	5	17	0

**TABLE 3:** Quantification of oerlap of GFP and Timeless (TIM) staining in sNPF‐GAL4+40xUAS‐mCD8::GFP + CRY‐GAL80 flies.

Cell type	Animals	Countable hemispheres	Total TIM+ cells	Cells with GFP overlap
Male				
LNds	4	7	42	0
s‐LNvs	4	7	34	0
l‐LNvs	4	7	23	0
Female				
LNds	2	4	16	1
s‐LNvs	2	3	19	0
l‐LNvs	2	4	12	0

### 3.5 Examination of the Roles of MB Neurons in the Changes in Rest/Sleep Behavior Following sNPF Neuron Activation

To examine the role of MB neurons in the sNPF‐mediated sleep response, an MB‐GAL80 transgene was used. A sleep experiment was carried out on sNPF > CHR, sNPF > CHR + MB‐GAL80, and MB‐GAL80 Alone female flies as in Figure 2, with the exception that the hour of red‐light stimulation was given at CT7 instead of CT8.

**FIGURE 5:**
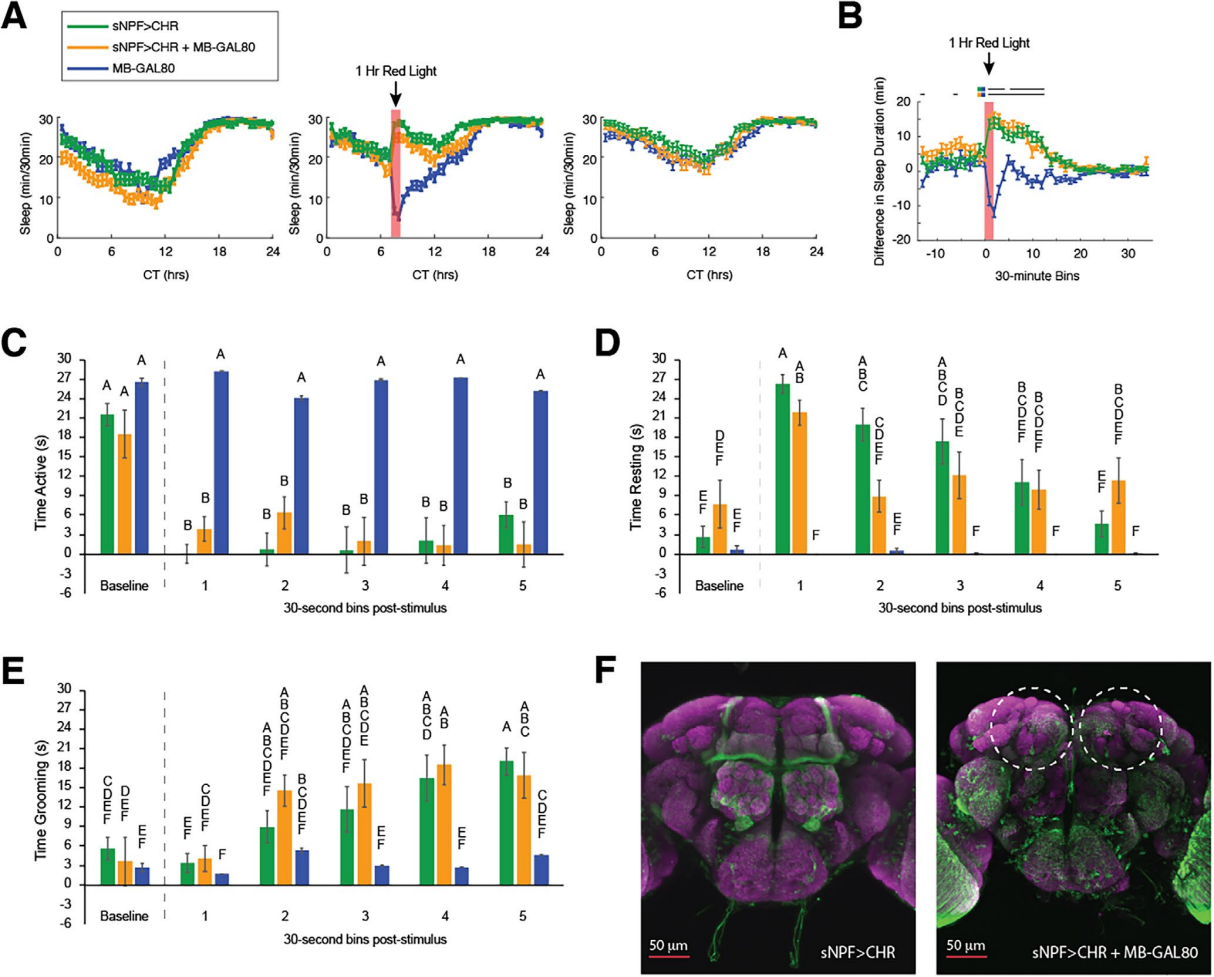
sNPF neurons in the mushroom bodies are not responsible for optogenetic sleep induction. (A, B) Data from a sleep study with a similar timeline to that shown in Figure 2. (A) Daily amounts of sleep in 30‐min bins across the three recording days, with 1 h of red light applied at CT7 on Day 2. (B) Sleep subtraction plot showing differences between sleep on the stimulation day minus the baseline day. Red‐light stimulation significantly increased sleep in both the sNPF > CHR and sNPF > CHR + MB‐GAL80 groups compared with the MB‐GAL80 control group for several hours following stimulation. (C–E) Data from an acute stimulation and video recording study with an identical timeline to that shown in Figure 3. Figures depict the time spent actively walking (C), resting (D), and grooming (E) during the 30‐s baseline and five post‐stimulation bins. Letters above bars represent significance groups—any groups that do not share a letter are significantly different from each other. (F) Confocal images (max projections) of brains from a sNPF > CHR (left) and sNPF > CHR + MB‐GAL80 (right) fly. The location of Chrimson‐mVenus expression was localized with anti‐GFP staining (green) on a background of nc82 staining (magenta). The dashed circles indicate the lack of GFP expression in the mushroom bodies. All graphs depict means ± SEMs. *N*'s for the sleep experiment were 77 for the sNPF > CHR group, 79 for the sNPF > CHR + MB‐GAL80 group, and 83 for the MB‐GAL80 group. *N*'s for the acute stimulation data were 10 for the sNPF > CHR group, nine for the sNPF > CHR + MB‐GAL80 group, and nine for the MB‐GAL80 group. All behavioral data were from female flies.

Both sNPF > CHR and sNPF > CHR + MB‐GAL80 flies exhibited a long‐lasting increase in sleep during and following the red‐light stimulation (Figure 5A,B). Analysis of the subtraction of sleep during each 30‐min period of the stimulation day minus the sleep during the same period of the baseline day showed a significant interaction between genotype and time bin (*F*(94, 11,092) = 10.87, *p* < 0.0001). Post hoc testing based on the subtraction analysis revealed that sNPF > CHR and sNPF > CHR + MB‐GAL80 flies were both significantly different from MBGAL80 controls during the red‐light stimulation and for 5 h afterward (Figure 5B). The only difference between the responses in the two experimental groups was one bin 1.5 h after the stimulus when the sleep subtraction was significantly different from controls for the sNPF > CHR + MB‐GAL80 group but not for the sNPF > CHR group. sNPF > CHR + MB‐GAL80 flies also had significantly greater sleep subtractions than MBGAL80 controls during two bins prior to red‐light stimulation. No significant differences were observed between sNPF > CHR + MB‐GAL80 and sNPF > CHR flies. On the recovery day, all groups of flies showed equivalent levels and timing of sleep.

When comparing sleep subtraction data during and after the red‐light stimulus with sleep during the pre‐stimulation 30‐min bin for each genotype, sNPF > CHR flies exhibited significantly higher levels of sleep during the two stimulation bins and for one subsequent bin. The sNPF > CHR + MB‐GAL80 flies showed significantly higher levels of sleep for the stimulation bins and for the following six bins. In contrast, MBGAL80 controls showed *lower* levels of sleep during the stimulus period and for one subsequent bin compared to the pre‐stimulation bin.

In our MB‐GAL80 sleep experiments, we also examined the effects of sNPF neuron activation on sleep/wake architecture (Table S1 and Figure S1). In these experiments, activation of all sNPF neurons produced changes to sleep/wake structure during the first 6‐h period following activation that were similar to those observed in the CRY‐GAL80 and PDF‐GAL80 experiments described above. These effects included increased total sleep time, decreased number of sleep episodes, and decreased Pwake (Figure S1D). The inclusion of the MB‐GAL80 transgene did not prevent any of these effects. Overall, these sleep studies suggest that sNPF neurons within the mushroom bodies are not critical for driving long‐lasting increases in sleep.

To examine the role of MB neurons in the acute effects of sNPF neuron activation, flies were videotaped for a 30‐s baseline period, during stimulation with red light for 5 s, and for an additional 2.5 min afterward, as described previously in Figure 3. We found significant interactions between genotype and time bin for time spent actively walking (*F*(10, 125) = 5.868, *p* < 0.0001) (Figure 5C), time spent resting (*F*(10, 125) = 5.617, *p* < 0.0001) (Figure 5D), and time spent grooming (*F*(10, 125) = 2.231, *p* < 0.02) (Figure 5E). Overall, flies in which all sNPF neurons were activated and flies in which all sNPF neurons except those in the MBs were activated had very similar behavioral patterns. Compared to the MBGAL80 Alone control group, neither sNPF > CHR nor sNPF > CHR + MB‐GAL80 had any significant alterations in time spent walking, resting, or grooming during the baseline period (Figure 5C–E). Following stimulation, both experimental groups had a strong reduction in active walking behavior for the remainder of the recording (Figure 4C). In the initial periods following red‐light stimulation, sNPF > CHR and sNPF > CHR + MB‐GAL80 flies spent the majority of their time resting, but as time progressed, resting time decreased (Figure 5D) and both groups spent more and more time grooming (Figure 5E). Both experimental groups also exhibited qualitatively similar uncoordinated movement responses during the light stimulation, involving twitching of the limbs and falling over. As expected, MB‐GAL80 Alone control flies did not show any significant changes in behavior during or after red‐light stimulation. Confocal imaging confirmed that sNPF‐GAL4‐driven expression of Chrimson in the mushroom bodies was eliminated by the addition of MB‐GAL80 (Figure 5F).

## 4 Discussion

The primary goal of this study was to determine which population(s) of sNPF‐expressing neurons were responsible for increases in sleep following sNPF neuron activation. Through GAL80‐mediated exclusion of optogenetic stimulation of specific sNPF neuron populations, we were able to show that sNPF/CRY‐positive, PDF‐negative clock neurons contribute to the late phase of the increase in sleep, whereas sNPF/PDF‐positive neurons and sNPF‐expressing neurons in the mushroom bodies (MB) do not. In experiments using CRY‐GAL80, in which all neurons targeted by our sNPF‐GAL4 driver except for those also expressing Cryptochrome (CRY) were activated optogenetically, we observed an initial increase in sleep upon red light stimulation, but this increase did not persist for nearly as long as it did in flies in which all sNPF neurons were activated (Figure 2). Changes to sleep architecture that were observed following activation of all sNPF neurons, such as increased sleep episode duration and lowered probability of transitioning from sleep to wake (Pwake), were also not observed when CRY‐GAL80 was included (Figure S1). This suggests that a population of sNPF/CRY‐positive clock neurons are crucial for the persistence of the sleep increase induced by sNPF neuron activation. Based on the known expression patterns of both sNPF and CRY, this initially pointed towards a role in sleep promotion for the small ventrolateral neurons (s‐LNvs) or a subset of the dorsolateral neurons (LNds) [34]. When we used PDF‐GAL80, which represses Chrimson expression within PDF‐expression neurons, including the s‐LNvs, sleep induction was identical to when activating all sNPF neurons (Figure 2). Taken together, our behavioral results using CRY‐GAL80 and PDF‐GAL80 suggest that sNPF‐expressing LNd clock neurons are important for generating the long‐lasting sleep increase when all sNPF neurons are activated. Interestingly, a previous study showed that sleep promotion due to thermogenetic activation of sNPF neurons was repressed by GAL80s targeting cholinergic neurons [45]. The sNPF‐expressing LNds are also predicted to be cholinergic and express CRY [34]. Both of these previous reports are thus consistent with our current findings.

We used immunostaining to confirm the efficacy of our GAL80 transgenes in repressing sNPF‐GAL4‐mediated gene expression. Based on previous literature, we expected that our sNPF‐GAL4 driver's expression pattern would include populations of MB Kenyon cells [39], as well as 2 TIM‐positive LNds and about 4 TIM/PDF‐positive s‐LNvs per hemisphere [34]. We found that the sNPF‐GAL4 driver did target the MBs as expected, which was prevented effectively by MB‐GAL80 (Figures S2 and 5). However, we were surprised to find that, in sNPF > Chrimson flies, we only observed occasional expression of Chrimson‐mVenus in TIM‐labeled LNds, and never saw expression in s‐LNvs (Figure S2). Using anti‐GFP staining to visualize the mVenus tag on the Chrimson protein was likely not strong enough to see neurons that were weakly targeted by the sNPF‐GAL4 driver. When we switched to 40xUAS‐mCD8::GFP to enhance the fluorescence signal, we did observe some GFP‐expressing LNds and s‐LNvs, but still not consistently, and more in males than in females (Table 2).

Although sNPF‐GAL4‐driven expression of Chrimson and GFP was observed less reliably in these cell types than anticipated, it is important to note that virtually all expression in the LNds and s‐LNvs was eliminated from the sNPF‐GAL4 expression pattern with the addition of CRY‐GAL80 (Figure 4 and Table 3). These results suggest that (1) the sNPF‐GAL4 driver does in fact target the LNds and s‐LNVs, at least in males, but at low levels that are only occasionally detectable, and (2) that CRY‐GAL80 is generally effective at preventing sNPF‐GAL4‐mediated expression within CRY‐positive clock neurons. The PDF‐GAL80 repressor clearly prevented PDF‐GAL4‐driven expression of GFP in both the l‐LNvs and s‐LNvs (Figure S4), demonstrating that PDF‐GAL80 was capable of preventing any sNPF‐GAL4‐driven expression of Chrimson in the LNvs. And yet PDF‐GAL80 did not impact the behavioral sleep response to optogenetic activation of sNPF‐GAL4 neurons, suggesting that the s‐LNvs are not a critical driver of the increase in sleep observed when sNPF‐GAL4 neurons are activated. Combined with our behavioral findings, these imaging results do support the idea that sNPF+ LNds could be responsible for sleep promotion when sNPF‐GAL4 neurons are activated—but they leave open the possibility that other sNPF+/CRY+ cells might play a role as well. Indeed, probing a single‐cell transcriptomics dataset from Davie et al. [59] revealed that there are far more sNPF+/CRY+/TIM− cells than sNPF+/CRY+/TIM+ cells, the latter of which should include LNds and s‐LNvs (Table S2). In addition, the CRY‐GAL80 transgene could have off‐target effects in sNPF+ neurons that do not naturally express CRY. These cells could also then be contributors to the sleep‐promoting effect of activating sNPF neurons.

To further assess whether excluding neuronal subsets using GAL80 caused any differences in initial behavioral responses to sNPF neuron activation, we used video analysis to examine the acute behavioral effects of just 10 s of sNPF neuron activation. We found that using GAL80 to prevent CRY‐positive or PDF‐positive neurons from being activated did not largely affect the flies' initial reactions to the light stimulation, or the increase in rest observed immediately after stimulation had ceased. However, we did find that, compared with flies in which all sNPF neurons were activated, their increase in rest tapered off more rapidly during the ensuing minutes following stimulation, and was replaced by grooming behavior. To anthropomorphize, it seems that these flies were gradually waking up faster than flies where all sNPF neurons were activated, and were starting to dust themselves off as they did so. This result for CRY‐GAL80 was consistent with what we had observed for sleep in the longer‐term activation experiments, where the increase in sleep tapered off more rapidly when CRY‐positive neurons were excluded. However, it was surprising to see a similar effect for PDF‐GAL80, since excluding PDF‐positive neurons did not have any effect on the sleep increase observed in the longer‐term activation experiments. This could indicate that PDF‐expressing neurons play at least a minor role in contributing to the short‐term behavioral effects of sNPF neuron activation.

Activation of only non‐MB sNPF neurons had similar acute and long‐term behavioral effects as activating all sNPF neurons, suggesting that MB neurons are not involved in the sleep response to sNPF neuron activation. This finding is in line with a previous study that used thermogenetics to activate sNPF neurons in combination with various GAL80 lines to limit expression patterns, including MB‐GAL80 [45]. sNPF was previously reported to be expressed in a large number of Kenyon cells within the mushroom bodies [33, 39], and we also observed that the sNPF‐GAL4 driver targeted four clusters of those cells (Figure 4). But sNPF within that region has been previously reported to play a role in olfactory memory [62]. Thus, it is possible that sNPF within the mushroom body primarily plays a role in behaviors other than sleep.

As we had observed previously [44], the most immediate response to sNPF neuron activation was motor discoordination, which ended as soon as the red‐light stimulus was turned off (see Video S1). This was followed by a period of increased rest, sometimes accompanied by lesser increases in grooming behavior (see Video S2). All of these responses still occurred with the addition of each of the GAL80 transgenes, suggesting that these responses are due to additional populations of neurons. The fact that the addition of CRY‐GAL80 reduced the duration of the sleep increase following sNPF neuron activation, but did not affect these earlier behavioral responses suggests that the long‐lasting increase in sleep is not simply a consequence of having undergone the immediate motor response. Future studies should endeavor to identify the neural pathways that are responsible for the more immediate reactions to sNPF neuron activation. sNPF has been reported to play a role in sensory‐motor feedback underlying nociceptive responses [63]. Thus, these sNPF‐expressing peripheral neurons may be mediating the immediate motor responses. The identity of neurons driving the early phase of increased sleep is still unknown.

This report has focused on the behavioral effects of sNPF neuron activation in female flies, due to the fact that male flies slept a large percentage of the time. This led to a ceiling effect, in which the high sleep levels prevented any further increase upon red‐light stimulation. The high level of sleep in male flies may be due to the Chrimson channel being leaky, allowing for ions to pass through the channel even without red‐light stimulation, thus activating the neurons where it is expressed without temporal specificity. While it is not apparent why this effect would be more prevalent in males but not females, it may be possible to circumvent this issue in the future by expressing Chrimson at lower levels. In our CRY‐GAL80 experiments, we observed a reduction in baseline sleep in comparison with sNPF + Chrimson flies that had been crossed with w‐ (Figure 2). It is worth noting that this did not occur with PDF‐GAL80 or MB‐GAL80. In addition, CRY‐GAL80 crossed with w‐ had similar baseline sleep levels to sNPF + Chrimson flies. Thus, the lowered sleep in the combined line cannot be explained by a different genetic background in the CRY‐GAL80 line. Instead, it could be explained by leaky Chrimson‐mediated activation of non‐CRY neurons targeted by the sNPF‐GAL4 driver. For example, if there are wake‐promoting, CRY‐negative neurons, then in the sNPF > CHR + CRY‐GAL80 flies, those neurons may be activated non‐specifically at a low level, resulting in reduced sleep. Whereas in sNPF > CHR flies, sleep‐promoting, CRY‐positive neurons might also be activated, counteracting that effect.

In the future, further refining of sNPF neuron activation will be necessary to fully characterize its roles in behavioral regulation. Given that sNPF is expressed in a large array of neurons [33], additional refinement of neuronal targeting to activate more selective sets of neurons, using split GAL4 lines [64, 65, 66] or the SPARC technique [67], might be able to provide more information about the roles of specific neurons in the sNPF‐mediated sleep response. Given that CRY has been previously shown to play a role in the entrainment of the circadian clock to light [36], it may be valuable in the future to record activity levels for a longer duration to examine if CRY‐positive neurons are responsible for any circadian shifts due to sNPF neuron activation. In addition to further refining neuronal activation, it will be of interest to identify the pathways downstream of sNPF signaling that are responsible for regulating sleep. For example, one could use methods such as RNA interference (RNAi) to downregulate the expression of the sNPF receptor in specific neuronal populations to determine which targets of sNPF are critical for the increase in sleep. Furthermore, RNAi could also be used to knock down expression of sNPF itself, thus distinguishing the effects of sNPF from effects of its co‐transmitters, since optogenetic activation can cause release of all transmitters in a given neuron.

This study has shown that various populations of sNPF‐expressing neurons contribute differently to the short‐term rest and long‐term sleep responses to sNPF neuron activation in *Drosophila*. In particular, CRY‐positive neurons within the sNPF‐GAL4 expression pattern appear to be critical for long‐lasting increases in sleep. These findings are consistent with the suggestion that sNPF may have a variety of complex roles in the fly brain [33], opening up many more questions about the roles of this neuropeptide in regulating behavior. Following this line of research further by more thoroughly characterizing the roles of sNPF in varying neuron populations will allow for a better understanding of both sleep and neuropeptide regulation more broadly. Given the homology of the sNPF receptor to the receptor for NPY in mammals [32], understanding the sNPF system could provide important insight into the NPY system in humans, allowing for better understanding of sleep in humans and development of better treatments for sleep disorders.

## Conflicts of Interest

The authors declare no conflicts of interest.

## Supporting information


**Figure S1.** Sleep/wake architecture during the 24‐h period following sNPF neuron activation. Total sleep duration, sleep episode number, mean sleep episode duration, activity per minute awake, Pdoze, and Pwake were calculated across four 6‐h bins starting at the onset of optogenetic stimulation in each sleep experiment from Figures 2 and 5. (A) Data from 1‐h stimulation CRY‐GAL80 experiments. (B) Data from 15‐min stimulation CRY‐GAL80 experiments. (C) Data from 1‐h stimulation PDF‐GAL80 experiments. (D) Data from 1‐h MB‐GAL80 experiments. All graphs depict means ± SEM, and significant differences were calculated by mixed‐model ANOVAs with Genotype as a between‐subject factor and Bin Number as a within‐subject factor, followed by Tukey post hoc tests. *Significant difference relative to the GAL80 control flies; and ^$^Significant difference between sNPF > CHR flies and sNPF > CHR + GAL80 flies. Table S1 contains the ANOVA outputs for the statistical analysis.

**Figure S2.** Effects of CRY‐GAL80 and PDF‐GAL80 on sNPF‐GAL4‐mediated expression of Chrimson. Anti‐GFP immunostaining (cyan) detected the presence of the CsChrimson‐mVenus protein driven by sNPF‐GAL4. In (A) and (C), brains were also stained against Timeless (TIM; yellow) to detect clock neurons with nc82 (magenta) as an anatomical background. In (B) and (D), brains were also stained against PDF (yellow) to detect ventrolateral neurons (LNvs), with NCAD (magenta) as an anatomical background. (A) Confocal images from an sNPF > CHR fly brain co‐stained for TIM. (Ai) Max projection image of a brain hemisphere. The dashed circles represent, from dorsal to ventral, the mushroom body, the dorsolateral clock neurons (LNds), and the LNvs. (Aii–iv) Cell body scans of the LNds for all signals (ii), TIM alone (iii), and Chrimson alone (iv) show that there was overlap of Chrimson and TIM in two of the LNds in the (arrows). (Av–vii) Cell body scans of the LNvs for all signals (v), TIM alone (vi), and Chrimson alone (vii) show that there was no overlap of Chrimson and TIM in the LNvs. (B) Confocal images from an sNPF > CHR fly brain co‐stained for PDF. (Bi) Max projection image of a brain hemisphere. The dashed circle represents the LNvs. (Bii–iv) Cell body scans of the LNvs for all signals (ii), PDF alone (iii), and Chrimson alone (iv) show that there was no overlap of Chrimson and PDF in the LNvs. (C) Confocal images from an sNPF > CHR + CRY‐GAL80 fly brain co‐stained for TIM. (Ci) Max projection image of a brain hemisphere. The dashed circles, from dorsal to ventral, represent the LNds and LNvs. (Cii–iv) Cell body scans of the LNds for all signals (ii), TIM alone (iii), and Chrimson alone (iv) show that there was no overlap of Chrimson and TIM in the LNds in the presence of CRY‐GAL80. (Cv–vii) Cell body scans of the LNvs for all signals (v), TIM alone (vi), and Chrimson alone (vii) show that there was no overlap of Chrimson and TIM in the LNvs in the presence of CRY‐GAL80. (D) Confocal images from an sNPF > CHR + PDF‐GAL80 fly brain co‐stained for PDF. (Di) Max projection image of a brain hemisphere. The dashed circle represents the LNvs. (Dii–iv) Cell body scans of the LNvs for all signals (ii), PDF alone (iii), and Chrimson alone (iv) show that there was no overlap of Chrimson and PDF in the LNvs in the presence of PDF‐GAL80. Puncta in TIM‐stained brains seem to be located along TIM‐positive projections. Some similar puncta can be seen in the PDF‐stained brains, especially in the example from the sNPF > CHR fly. All images were taken with a ×40 objective.

**Figure S3.** Effects of CRY‐GAL80 and PDF‐GAL80 on sNPF‐GAL4‐mediated expression of Chrimson within the ventral nerve cord (VNC). Anti‐GFP immunostaining (cyan) detected the presence of the CsChrimson‐mVenus protein driven by sNPF‐GAL4. In (A) and (C), VNCs were also stained against Timeless (TIM; yellow) to detect clock neurons with nc82 (magenta) as an anatomical background. In (B) and (D), VNCs were also stained against PDF (yellow) to detect ventrolateral neurons (LNvs), with NCAD (magenta) as an anatomical background. Each of the main VNC images consists of two ×40 images that were manually aligned to provide a full view of the VNC. Each VNC came from the same example animal for each group whose brain is shown in Figure 4, except for the TIM‐stained VNC from the sNPF > CHR group. (A) Confocal images from an sNPF > CHR fly VNC co‐stained for TIM. (Ai) Max projection images of the VNC. (Aii–iv) Cell body scans of the abdominal TIM‐stained cells for all signals (ii), TIM alone (iii), and Chrimson alone (iv) show that there was no overlap of Chrimson and TIM in these cells. (B) Confocal images from an sNPF > CHR fly VNC co‐stained for PDF. (Bi) Max projection images of the VNC. (Bii–iv) Cell body scans of the abdominal PDF‐stained cells for all signals (ii), PDF alone (iii), and Chrimson alone (iv) show that there was no overlap of Chrimson and PDF in these cells. (C) Confocal images from an sNPF > CHR + CRY‐GAL80 fly VNC co‐stained for TIM. (Ci) Max projection images of a VNC. (Cii–iv) Cell body scans of the abdominal TIM‐stained cells for all signals (ii), TIM alone (iii), and Chrimson alone (iv) show that there was no overlap of Chrimson and TIM in these cells in the presence of CRY‐GAL80. (D) Confocal images from an sNPF > CHR + PDF‐GAL80 fly VNC co‐stained for PDF. (Di) Max projection images of a VNC. (Dii–iv) Cell body scans of the abdominal PDF‐stained cells for all signals (ii), PDF alone (iii), and Chrimson alone (iv) show that there was no overlap of Chrimson and PDF in these cells in the presence of PDF‐GAL80. All images were taken with a ×40 objective.

**Figure S4.** Efficacy of PDF‐GAL80 to block PDF‐GAL4‐mediated expression of GFP within the brain. Confocal imaging showing anti‐GFP immunostaining (green) driven by PDF‐GAL4, and anti‐PDF staining (red), with overlap showing up as yellow. (A) Brains from flies containing PDF‐GAL4 and UAS‐mCD8::GFP transgenes were imaged. Endogenous PDF protein and PDF‐GAL4‐driven GFP colocalized in identifiable large and small ventrolateral neurons (l‐LNvs and s‐LNvs). (B) The addition of PDF‐GAL80 completely repressed GFP expression in both l‐LNvs and s‐LNvs.

**Table S1.** ANOVA results for sleep architecture analysis across 24 h following optogenetic stimulation (supports Figure S1). Yellow highlighting indicates significant *p* values (< 0.05) and orange highlighting indicates trends (0.05–0.10). Only Genotype × Bin interactions that reached significance were followed by Tukey post hoc tests.

**Table S2.** Overlap of sNPF, Timeless, and Cryptochrome based on transcriptomics data. The SCOPE dataset from Davie et al. [1] was probed for cells expressing short neuropeptide F (sNPF), Timeless (TIM), and/or Cryptochrome (CRY) in 6‐ and 9‐day‐old male and female flies. The dataset predicts that not all sNPF+/CRY+ cells also express TIM, and also predicts that the number of cells expressing all three genes declines between ages 6 and 9.

**Video S1.** Example of baseline behavior and response to brief sNPF neuron activation. Shown is an sNPF > CHR + MB‐GAL80 fly, during a portion of the 30‐s baseline period, during a 5‐s red‐light pulse, and during the immediate post‐stimulation period. The fly spent the baseline walking, experienced an immediate uncoordinated response during the red‐light stimulation, and then recovered quickly afterward. This pattern was consistent for all groups of flies in which sNPF neurons were activated, regardless of the presence of a GAL80 transgene.

**Video S2.** Example of behavior minutes after brief sNPF neuron activation. Shown is an sNPF > CHR + MB‐GAL80 fly (the same as in Video S1), during the last portion of the 2.5‐min post‐stimulation period following a 5‐s red‐light pulse. During the post‐stimulation period, flies spent most of their time either resting or grooming, the latter of which is emphasized in this video. Time spent walking was greatly reduced.

**Video S3.** Example of behavior before, during, and after prolonged sNPF neuron activation. Shown is an sNPF > CHR fly. The video is spliced once to show behavior during a portion of the 30‐s baseline period and during the first period of a 15‐min red light pulse, and then during the last period of the same red‐light pulse and during the immediate post‐stimulation period. The nature of the uncoordinated response during red‐light stimulation changed from beginning to end, but flies were still unable to move effectively at the end of the 15‐min stimulation period. However, they were able to recover quickly following stimulation.

**Video S4.** Example of behavior before, during, and after prolonged sNPF neuron activation in the presence of CRY‐GAL80. Shown is an sNPF > CHR + CRY‐GAL80 fly. The video is spliced once to show behavior during a portion of the 30‐s baseline period and during the first period of a 15‐min red light pulse, and then during the last period of the same red‐light pulse and during the immediate post‐stimulation period. The pattern of responses to a longer duration of red‐light stimulation was very similar flies in which CRY‐positive neurons were excluded from being activated as in flies in which all sNPF neurons were activated (Video S3).

**Video S5.** Scan through the brain of an sNPF > CHR fly stained to show expression of Chrimson, Timeless, and Bruchpilot. The video represents a sequence of optical sections taken with confocal microscopy of the brain of an sNPF > CHR fly, progressing from anterior to posterior. Shown are anti‐GFP labeling for Chrimson‐mVenus (cyan), localization of Timeless (yellow) and Bruchpilot to show neuropil (magenta). At the 5‐second mark, a dorsolateral (LNd) clock cell can be seen that is co‐stained for Chrimson and Timeless.

**Video S6.** Scan through the anterior portion of the ventral nerve cord of an sNPF > CHR fly stained to show expression of Chrimson, Timeless, and Bruchpilot. The video represents a sequence of optical sections taken with confocal microscopy of the anterior portion of the ventral nerve cord (VNC) of an sNPF > CHR fly, progressing from ventral to dorsal. Shown are anti‐GFP labeling for Chrimson‐mVenus (cyan), localization of Timeless (yellow) and Bruchpilot to show neuropil (magenta). There is no clear co‐staining for Chrimson and Timeless.

**Video S7.** Scan through the posterior portion of the ventral nerve cord of an sNPF > CHR fly stained to show expression of Chrimson, Timeless, and Bruchpilot. The video represents a sequence of optical sections taken with confocal microscopy of the posterior portion of the ventral nerve cord (VNC) of an sNPF > CHR fly (the same as in Video S6), progressing from ventral to dorsal. Shown are anti‐GFP labeling for Chrimson‐mVenus (cyan), localization of Timeless (yellow) and Bruchpilot to show neuropil (magenta). There is no clear co‐staining for Chrimson and Timeless.

## Data Availability

The data that support the findings of this study are available from the corresponding author upon reasonable request.
