# Supplementary figures and images for: The Roles of Discrete Populations of Neurons Expressing Short Neuropeptide F in Sleep Induction in Drosophila melanogaster
Source: Genes Brain Behav. 2025 Feb 7;24(1):e70010. doi: 10.1111/gbb.70010 (PMC11804769; doi:10.1111/gbb.70010)

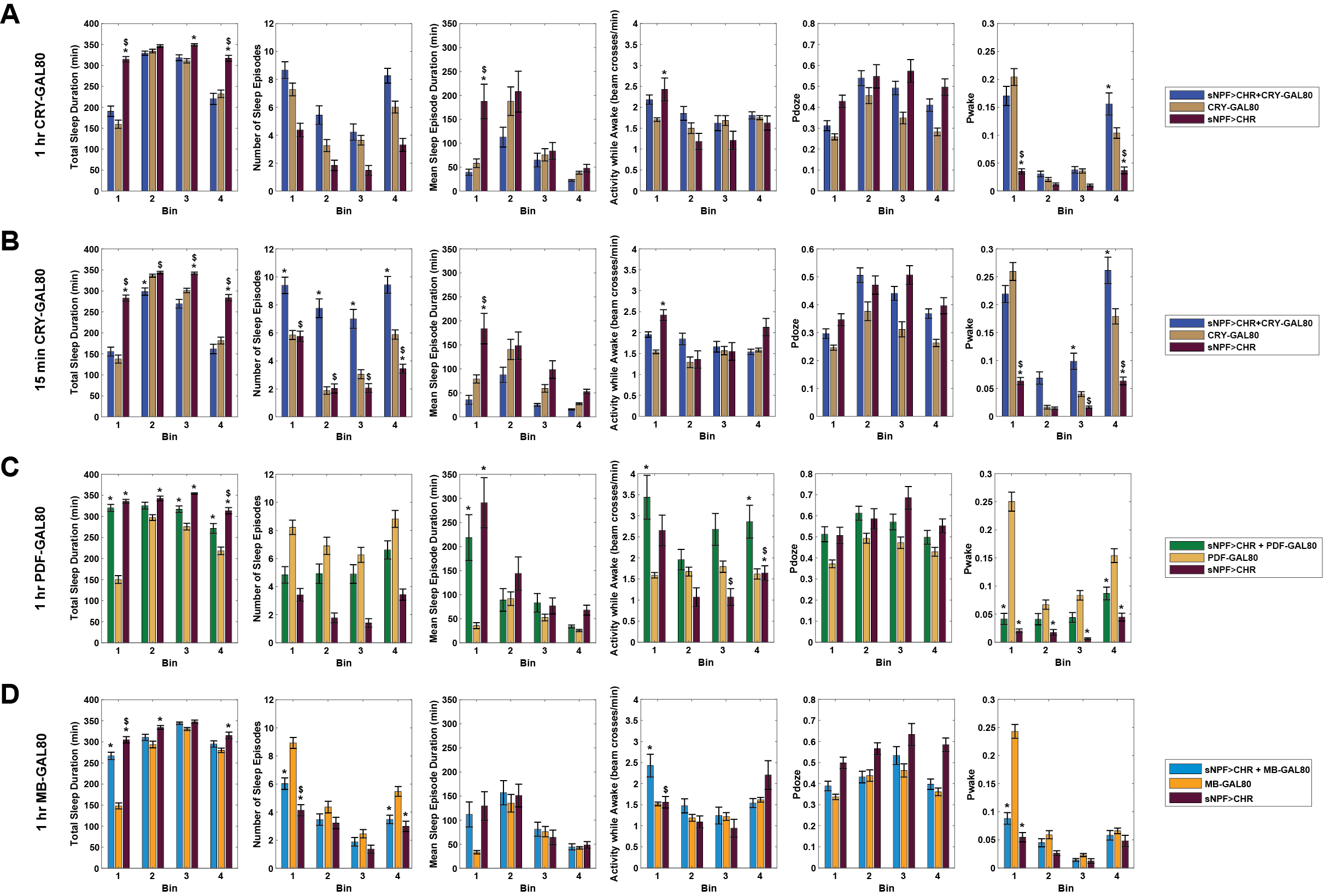

Supplement: Supplementary file 1 — Figure S1. Sleep/wake architecture during the 24‐h period following sNPF neuron activation. Total sleep duration, sleep episode number, mean sleep episode duration, activity per minute awake, Pdoze, and Pwake were calculated across four 6‐h bins starting at the onset of optogenetic stimulation in each sleep experiment from Figures 2 and 5. (A) Data from 1‐h stimulation CRY‐GAL80 experiments. (B) Data from 15‐min stimulation CRY‐GAL80 experiments. (C) Data from 1‐h stimulation PDF‐GAL80 experiments. (D) Data from 1‐h MB‐GAL80 experiments. All graphs depict means ± SEM, and significant differences were calculated by mixed‐model ANOVAs with Genotype as a between‐subject factor and Bin Number as a within‐subject factor, followed by Tukey post hoc tests. *Significant difference relative to the GAL80 control flies; and $Significant difference between sNPF > CHR flies and sNPF > CHR + GAL80 flies. Table S1 contains the ANOVA outputs for the statistical analysis. [file GBB-24-e70010-s008.png]

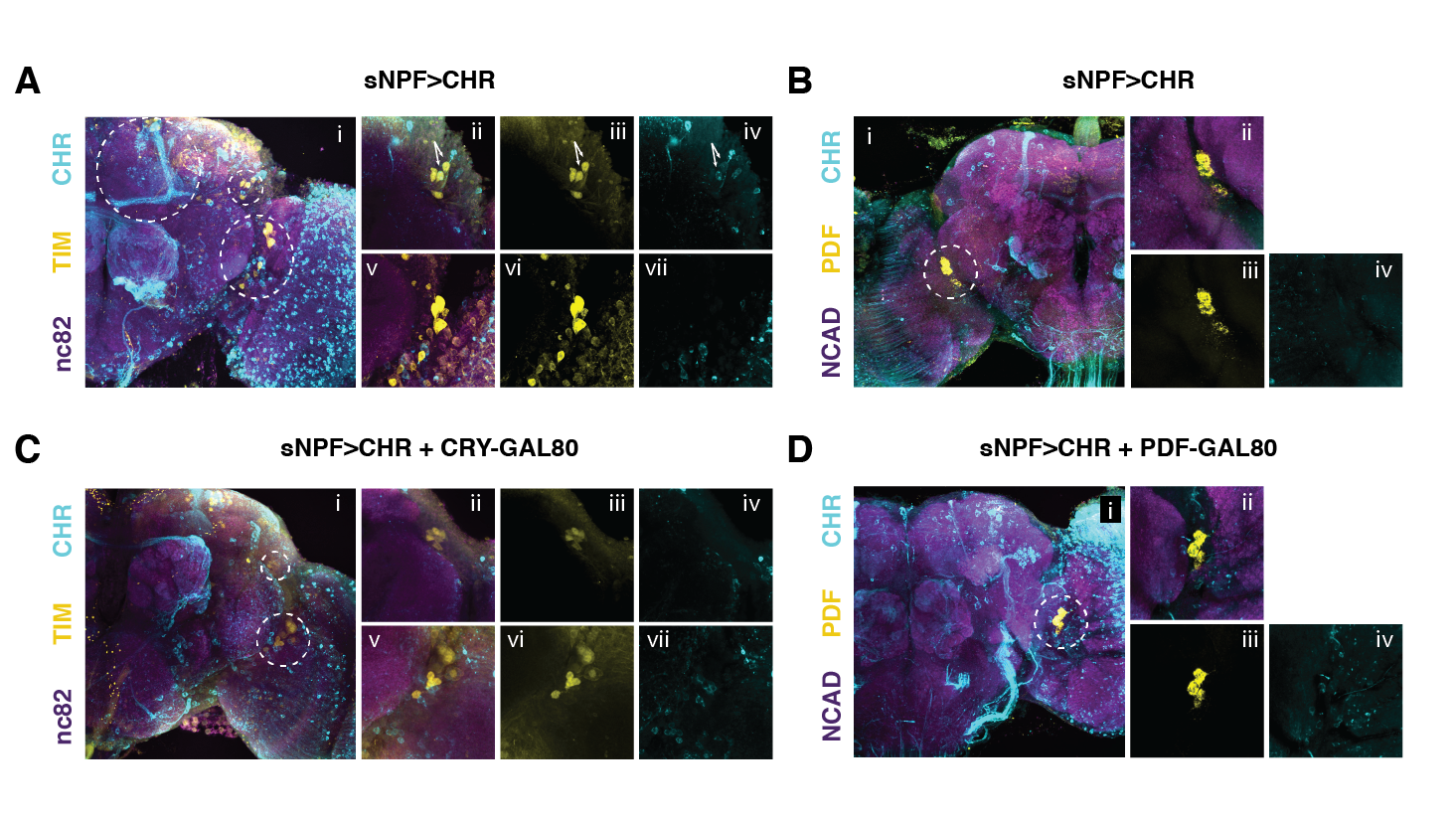

Supplement: Supplementary file 2 — Figure S2. Effects of CRY‐GAL80 and PDF‐GAL80 on sNPF‐GAL4‐mediated expression of Chrimson. Anti‐GFP immunostaining (cyan) detected the presence of the CsChrimson‐mVenus protein driven by sNPF‐GAL4. In (A) and (C), brains were also stained against Timeless (TIM; yellow) to detect clock neurons with nc82 (magenta) as an anatomical background. In (B) and (D), brains were also stained against PDF (yellow) to detect ventrolateral neurons (LNvs), with NCAD (magenta) as an anatomical background. (A) Confocal images from an sNPF > CHR fly brain co‐stained for TIM. (Ai) Max projection image of a brain hemisphere. The dashed circles represent, from dorsal to ventral, the mushroom body, the dorsolateral clock neurons (LNds), and the LNvs. (Aii–iv) Cell body scans of the LNds for all signals (ii), TIM alone (iii), and Chrimson alone (iv) show that there was overlap of Chrimson and TIM in two of the LNds in the (arrows). (Av–vii) Cell body scans of the LNvs for all signals (v), TIM alone (vi), and Chrimson alone (vii) show that there was no overlap of Chrimson and TIM in the LNvs. (B) Confocal images from an sNPF > CHR fly brain co‐stained for PDF. (Bi) Max projection image of a brain hemisphere. The dashed circle represents the LNvs. (Bii–iv) Cell body scans of the LNvs for all signals (ii), PDF alone (iii), and Chrimson alone (iv) show that there was no overlap of Chrimson and PDF in the LNvs. (C) Confocal images from an sNPF > CHR + CRY‐GAL80 fly brain co‐stained for TIM. (Ci) Max projection image of a brain hemisphere. The dashed circles, from dorsal to ventral, represent the LNds and LNvs. (Cii–iv) Cell body scans of the LNds for all signals (ii), TIM alone (iii), and Chrimson alone (iv) show that there was no overlap of Chrimson and TIM in the LNds in the presence of CRY‐GAL80. (Cv–vii) Cell body scans of the LNvs for all signals (v), TIM alone (vi), and Chrimson alone (vii) show that there was no overlap of Chrimson and TIM in the LNvs in the presence of CRY‐GAL80. (D) C [file GBB-24-e70010-s001.png]

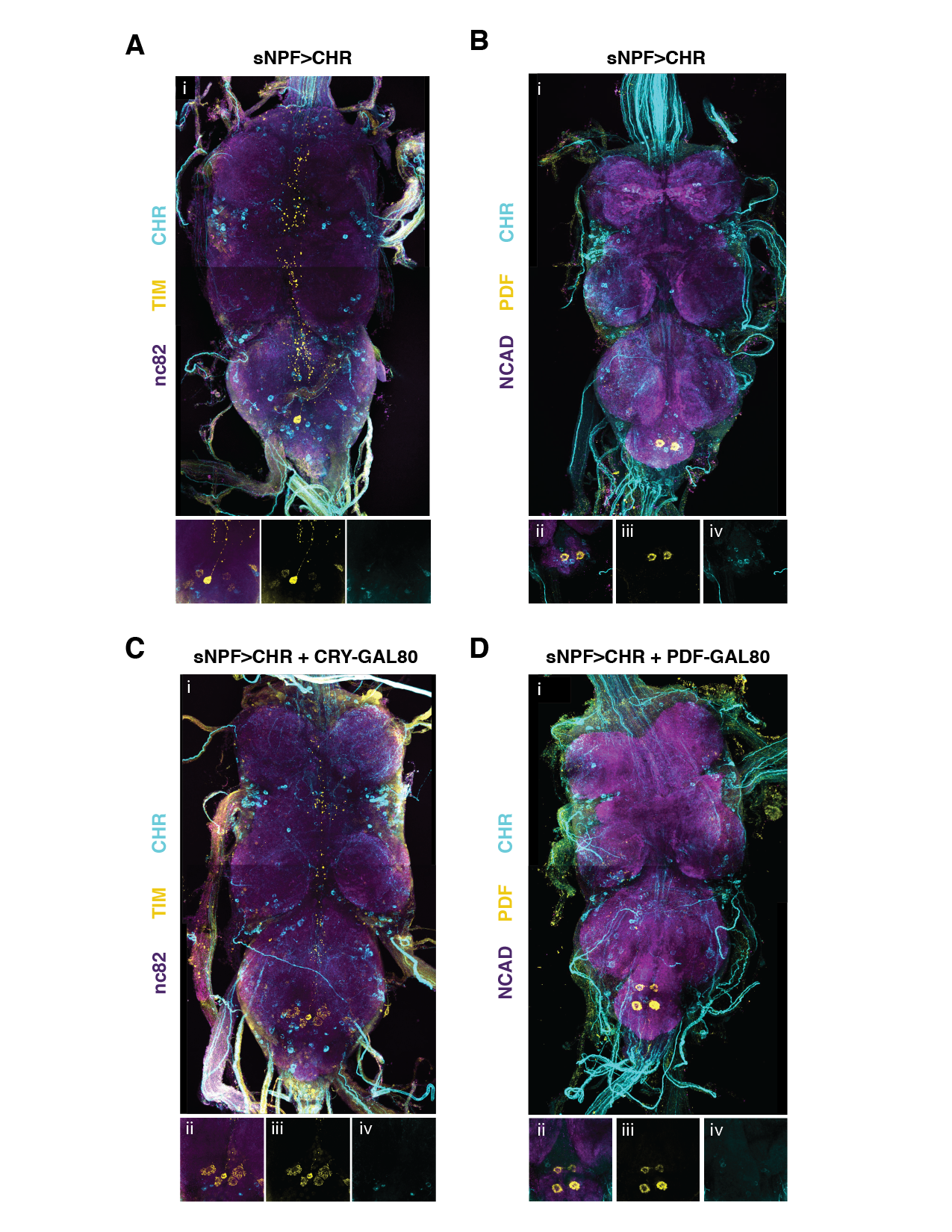

Supplement: Supplementary file 3 — Figure S3. Effects of CRY‐GAL80 and PDF‐GAL80 on sNPF‐GAL4‐mediated expression of Chrimson within the ventral nerve cord (VNC). Anti‐GFP immunostaining (cyan) detected the presence of the CsChrimson‐mVenus protein driven by sNPF‐GAL4. In (A) and (C), VNCs were also stained against Timeless (TIM; yellow) to detect clock neurons with nc82 (magenta) as an anatomical background. In (B) and (D), VNCs were also stained against PDF (yellow) to detect ventrolateral neurons (LNvs), with NCAD (magenta) as an anatomical background. Each of the main VNC images consists of two ×40 images that were manually aligned to provide a full view of the VNC. Each VNC came from the same example animal for each group whose brain is shown in Figure 4, except for the TIM‐stained VNC from the sNPF > CHR group. (A) Confocal images from an sNPF > CHR fly VNC co‐stained for TIM. (Ai) Max projection images of the VNC. (Aii–iv) Cell body scans of the abdominal TIM‐stained cells for all signals (ii), TIM alone (iii), and Chrimson alone (iv) show that there was no overlap of Chrimson and TIM in these cells. (B) Confocal images from an sNPF > CHR fly VNC co‐stained for PDF. (Bi) Max projection images of the VNC. (Bii–iv) Cell body scans of the abdominal PDF‐stained cells for all signals (ii), PDF alone (iii), and Chrimson alone (iv) show that there was no overlap of Chrimson and PDF in these cells. (C) Confocal images from an sNPF > CHR + CRY‐GAL80 fly VNC co‐stained for TIM. (Ci) Max projection images of a VNC. (Cii–iv) Cell body scans of the abdominal TIM‐stained cells for all signals (ii), TIM alone (iii), and Chrimson alone (iv) show that there was no overlap of Chrimson and TIM in these cells in the presence of CRY‐GAL80. (D) Confocal images from an sNPF > CHR + PDF‐GAL80 fly VNC co‐stained for PDF. (Di) Max projection images of a VNC. (Dii–iv) Cell body scans of the abdominal PDF‐stained cells for all signals (ii), PDF alone (iii), and Chrimson alone (iv) show that there was no overlap of Chrims [file GBB-24-e70010-s012.png]

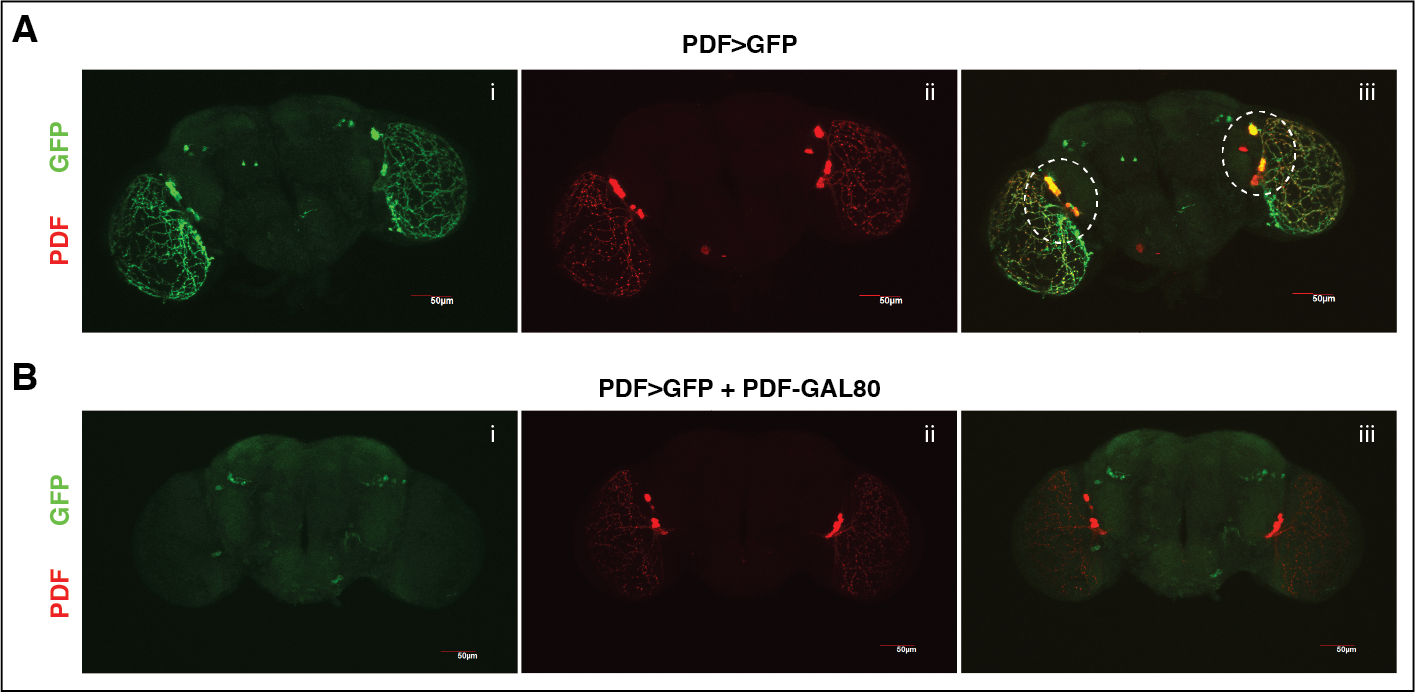

Supplement: Supplementary file 4 — Figure S4. Efficacy of PDF‐GAL80 to block PDF‐GAL4‐mediated expression of GFP within the brain. Confocal imaging showing anti‐GFP immunostaining (green) driven by PDF‐GAL4, and anti‐PDF staining (red), with overlap showing up as yellow. (A) Brains from flies containing PDF‐GAL4 and UAS‐mCD8::GFP transgenes were imaged. Endogenous PDF protein and PDF‐GAL4‐driven GFP colocalized in identifiable large and small ventrolateral neurons (l‐LNvs and s‐LNvs). (B) The addition of PDF‐GAL80 completely repressed GFP expression in both l‐LNvs and s‐LNvs. [file GBB-24-e70010-s006.png]
